# Wood Biomolecules as Agricultural Adjuvants for Effective Suppression of Droplet Rebound from Plant Foliage

**DOI:** 10.1002/advs.202416686

**Published:** 2025-03-10

**Authors:** Mamata Bhattarai, Hedar Al‐Terke, Kai Liu, Zhangmin Wan, Petri Kilpeläinen, Alistair W. T. King, Alexey Khakalo, Jiayun Xu, Chunlin Xu, Robin H. A. Ras, Bruno D. Mattos, Orlando J. Rojas

**Affiliations:** ^1^ Department of Bioproducts and Biosystems School of Chemical Engineering Aalto University Espoo FI‐00076 Finland; ^2^ Department of Applied Physics Aalto University Espoo FI‐00076 Finland; ^3^ Centre of Excellence in Life‐Inspired Hybrid Materials (LIBER) Aalto University Espoo FI‐00076 Finland; ^4^ Department of Chemical & Biological Engineering Department of Chemistry Department of Wood Science Bioproducts Institute The University of British Columbia Vancouver BC V6T 1Z3 Canada; ^5^ Production Systems Biomass Fractionation Technologies Natural Resource Institute Finland (LUKE) Viikinkaari 9 Helsinki 00790 Finland; ^6^ Bioinspired Materials Cellulose Coatings and Films VTT Technical Research Centre of Finland Ltd. Tietotie 4E Espoo FI‐02044 Finland; ^7^ Laboratory of Natural Materials Technology Åbo Akademi University Henrikinkatu 2 Turku FI‐20500 Finland

**Keywords:** adhesion, agrochemical spreading, crop protection, droplet bouncing, hemicelluloses, leaves, lignin

## Abstract

The agrochemical run‐off associated with crop control is an unintended consequence of droplet rebound from plant foliage, which negatively affects crop performance and the environment. This is most critical in water‐based formulations delivered on plant surfaces that are typically waxy and nonwetting. This study introduces an alternative to synthetic surfactants and high molecular weight polymers that are used as spreading agents for agrochemicals. Specifically, biopolymeric adjuvants (hemicelluloses and oligomeric lignin) extracted from wood by pressurized hot water are shown for their synergistic pinning capacity and surface activity that can effectively suppress droplet rebound from hydrophobic surfaces. Hemicellulose and lignin mixtures, alongside several model compounds, are investigated for understanding the dynamics of droplet impact and its correlation with biomacromolecule formations. The benefit of utilizing lean solutions (0.1 wt.% concentration) is highlighted for reducing droplet rebounding from leaves, outperforming synthetic systems in current use. For instance, a tenfold deposition improvement is demonstrated on citrus leaves, because of a significantly suppressed droplet roll‐off. These results establish the excellent prospects of wood extracts to improve crop performance.

## Graphical abstract

Lignin and hemicellulose are used to develop agricultural adjuvants to decrease agrochemical run‐off from foliage, by suppressing droplet rebound and enhancing adhesion of aqueous solutions on plant leaves. The wood components combine surface activity with surface pinning effects and to improve by tenfold the droplet rebound in citrus leaves, outperforming traditional surfactants and polymers used in the field.

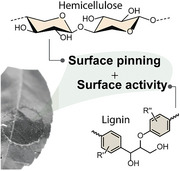

## 1 Introduction

Droplet deposition and spreading on hydrophobic surfaces are relevant to a variety of processes, such as spraying, inkjet printing, cleaning, and agrochemical application. The latter involves pesticides, insecticides, and herbicides, which are regularly applied by spraying to improve crop productivity. However, the retention of the active components from formulations designed for foliar surfaces is usually challenged by the waxy nature of leaves and their micro‐/nanostructured morphology.^[^ ^1^ ^]^ Bouncing, splashing, and sliding off of aqueous solutions carrying agrochemicals lead to inefficient retention of active ingredients, which directly and negatively impacts soil and aquatic ecosystems.^[^ ^2^ ^]^ Hence, ever‐evolving formulations are tested in a quest to improve droplet deposition on hydrophobic surfaces. For this purpose, wetting and adhesion can be tailored by surfactants, which reduce the surface tension of fluids.^[^ ^3^ ^]^ Preferred surfactants include those with low surface tension and that spread rapidly on hydrophobic surfaces.^[^ ^1^ ^]^ However, most surfactants are only effective above their respective critical micelle concentration, which colludes with efforts to reduce dosage levels.^[^ ^3^ ^]^ This is because high surfactant levels favor non‐specific binding and are toxic to plants and living species.^[^ ^2^ ^]^

Suppression of droplet rebounding and splashing can also be achieved by modifying the viscoelasticity of the fluid by adding high molecular weight polymers or colloids.^[^ ^1^ ^]^ Polyethylene oxide, polyacrylamide, and galactomannans (guar gum)^[^ ^4^ ^]^ have been shown to reduce rebounding by the effect of friction – generated from elongated polymer molecules – at the air‐liquid‐solid interface. They also result in significant energy dissipation from the impacting droplet,^[^ ^5^ ^]^ and act as viscosifyers.^[^ ^6^ ^]^ Recently, colloidal nanofibers based on cellulose^[^ ^7^ ^]^ and glycyrrhizic acid^[^ ^8^ ^]^ were tested to control the bouncing of droplets on plant leaves. In addition to the effect of nanofibers on friction and viscosity, their high aspect ratio induces pinning on substrates during droplet retraction. Silica nanoparticles are found to produce similar results but require high concentrations (>40 wt.%), given their limited ability to form interconnected networks.^[^ ^9^ ^]^ In general, the polymers and colloids considered so far offer some promise to control droplet impact behavior, but their effect on the overall viscosity of the suspensions/solutions limits their practical deployment, for instance by spraying. Non‐surface‐active polymers often need to be supplemented with surfactants to achieve the required wetting results.^[^ ^6^ ^]^ Overall, efforts in the area should consider costcompetitive, low‐viscosity, and efficient surface‐active adjuvants derived from non‐toxic and sustainable precursors.

We propose mildly surface‐active wood hemicelluloses and lignins suspended in water to reduce droplet rebounding from hydrophobic surfaces, which would ultimately promote better and more sustainable delivery of pesticide and foliage nutrients in plants. Hemicelluloses are heteropolysaccharides principally composed of xylans in hardwoods and galactoglucomannans in softwoods, whereas lignin are aromatic biomacromolecules based on *p‐*hydroxyphenyl, guaiacyl, and syringyl units. Combined, hemicelluloses and lignin make up to 60% of the dry mass of wood, offering great potential for wide application as agricultural adjuvants. Most relevant is the fact that large amounts of low molecular weight hemicelluloses and lignin (Mw <10 kDa) can be released from wood by simple hot‐water extraction.^[^ ^10^ ^]^ For example, 53–78 wt.% of hemicelluloses and 15–25 wt.% of lignin present in birch (hardwood) and spruce (softwood) sawdust can be isolated by pressurized hot‐water flow‐through extraction (PHWE). Moreover, water‐based extraction largely preserves the lignin structure of the precursor biomass.^[^ ^11^ ^]^ Hemicelluloses and lignin can independently co‐exist in the hydrolyzates, but can also assume covalently bound structures.^[^ ^12^ ^]^ These hemicellulose/lignin mixtures are naturally amphiphilic, i.e., they display a surfactant‐like behavior^[^ ^13^ ^]^ due to the presence of hydrophilic and partly hydrophobic moieties.^[^ ^14^ ^]^ Lignin alone is amphiphilic^[^ ^15^ ^]^ and form structured nanoparticles^[^ ^16^ ^]^ that can be used for emulsion stabilization.

**Figure 1:**
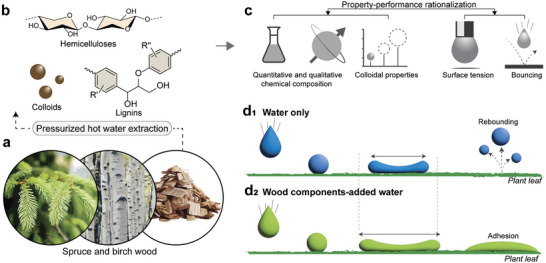
Wood‐based agricultural adjuvants. a) Spruce and birch wood sawdust are used to extract non‐cellulosic components by pressurized hot water extraction (PHWE). b) Hemicellulose and/lignin‐rich fractions (solubilized or colloidal forms) are released from wood according to the starting material and process conditions. c) Property‐performance was rationalized using extracted wood components characterized for their chemical and colloidal properties, surface tension, and bouncing performance. d_1,2_) Lean dispersions of the wood components in water improve spreading and adhesion on plant leaves (d_1_), whereas application of neat water results in significant droplet rebounding or splashing (d_2_).

Herein, we use pressurized hot‐water flow‐through extraction of birch and spruce sawdust (**Figure** **1**a) to generate aqueous fractions comprising hemicellulose and lignin, solubilized or as hydrocolloidal matter (Figure 1b). Our flow‐through process, differently than batch extraction, offers short hot water residence time in the reactor as fresh water is inserted and flown out. Whereas batch extraction tends to degrade hemicelluloses into furfurals, our process greatly preserves the structure of the extracted compounds because it simply cleaves hemicelluloses from wood biomass, carrying small lignin fragments.^[^ ^11^ ^]^ Both components, when co‐existing, form amphiphilic mixtures that are exploited herein as agricultural adjuvants. We investigate the impact of the hemicellulose and lignin composition on water droplet impact, wetting, and adhesion. The biobased extracts are characterized as far as their chemical composition, colloidal properties, and surface activity (Figure 1c), which are then used to identify the main factors affecting droplet impact dynamics and wetting behavior (Figure 1d). The droplet bouncing behavior is first studied using model hydrophobic surfaces (paraffin wax with a water contact angle, WCA = 110°), followed by application on citrus and cauliflower leaves (WCA, ca. 95° and 145°, respectively). Our experimental observations were supported by molecular dynamic simulations of interactions taking place between hemicellulose, lignin, and a hydrophobic model surface. Following proof‐of‐concept demonstrations we stablish a property‐performance correlation between the wood components and their effects on droplet rebound and pesticide efficiency setting the basis for the development of sustainable biobased adjuvants.

## 2 Results and Discussion

### 2.1 Physicochemical Properties and Surface Activity of PHWE Wood Extracts

**Figure 2:**
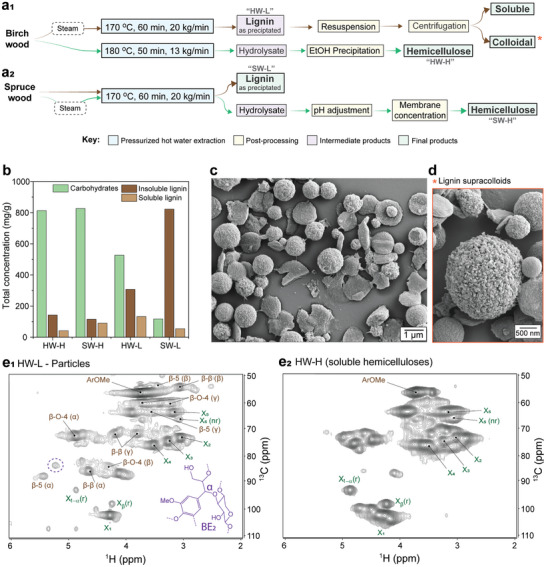
Release of hemicelluloses, lignin, and lignin‐carbohydrate complexes (LCCs) from soft‐ and hardwood by pressurize hot water extraction. Detailed processing strategies leading to lignin‐rich and hemicellulose‐rich fractions from a_1_) birch and a_2_) spruce wood. b) Overall chemical composition of the obtained fractions. c) Scanning electron microscopy images of lignin particles present in HW‐L (lignin‐rich fractions obtained from birch), detailing its d) supracolloidal morphology. Heteronuclear single quantum coherence (HSQC) nuclear magnetic resonance (NMR) spectra in e_1_) the particle fraction in HW‐L and e_2_) hemicellulose‐rich fraction from birch. Note: assignments and abbreviations in e_1_ and e_2_ are detailed in Table S2 (Supporting Information). Supporting NMR spectra are found in Figures S3 and S4 (Supporting Information).

Hemicellulose‐rich and lignin‐rich fractions were obtained from birch (hardwood) and spruce (softwood) by pressurized hot‐water extraction (PHWE) (**Figure** **2**a). The released fractions are referred to as HW‐H, HW‐L, SW‐H, and SW‐L (HW: hardwood, SW: softwood, H: hemicellulose, L: lignin). Lignin‐rich fractions derived from birch wood, given their intrinsically compositions variability, were further fractionated into a soluble and a colloidal fraction to assess their effect on droplet bouncing.

The PHWE process was designed to yield extracts rich in hemicellulose (HW‐H and SW‐H) and lignin (HW‐L and SW‐L). In the hemicellulose‐rich extracts, ≈82 wt.% of the dry mass of the extracted compounds were hemicelluloses with the remaining represented by lignin fractions and/or low molecular weight polyphenols. The lignin‐rich extracts contained from 40 to 85 wt.% of lignin with residual non‐cellulosic polysaccharides (primarily hemicelluloses) (Figure 2b and **Table** **1**; Table S1, Supporting Information). The lignin in such fractions was predominantly acid‐insoluble. By contrast, the hemicellulose‐rich fractions contained acid‐soluble lignin (Figure 2b). The released wood components were oligomeric in size, with an average molar mass between 1.7 and 5.5 kDa (Table 1). Lignin‐rich fractions were most in the form of particles (ca. 1–2 µm, Figure 2c and Figures S1 and S2, Supporting Information), owing to the poor solubility of lignin in water.^[^ ^17^ ^]^ Most particles were large and appeared to be formed by assemblies of small lignin nanoparticles (Figure 2d). They produced off‐white opaque aqueous suspensions at 1 wt.% and underwent rapid sedimentation. On the other hand, the hemicellulose‐rich fractions contained smaller particles (<0.65 µm) (Table 1) and formed colloidally stable suspensions. The hemicelluloses in the hemicellulose‐rich fractions were solubilized with a 80:20 ratio of soluble hemicellulose versus colloidal forms as verified with fractionation by stepwise ultracentrifugation (Figure S1, Supporting Information).^[^ ^18^ ^]^ Colloidal assemblies in the particle fraction of the HW‐L extract were enabled by glucuronoxylan‐lignin linkages supported by a higher content of guaiacyl (G) lignin units compared to its partitioned soluble fraction. G‐type monolignol units have more interacting sites compared to syringyl S type.^[^ ^19^ ^]^ On the other hand, the higher solubility of soluble HW‐L and that of HW‐H could be attributed to other branched structures from glucomannan‐lignin linkages and/or pectins,^[^ ^15^ ^]^ which are minor components in hardwoods.^[^ ^20^ ^]^

**Table 1:** Total carbohydrate and lignin content (mg g^−1^ dry extract), weight‐average molar mass (kDa), zeta potential (ζ, mV), average particle size (Z‐average), and surface tension of aqueous suspensions extracted by PHWE from hardwoods (HW) and softwoods (SW).

Sample	Total carbohydrates mg g^−1^	Total lignin mg g^−1^	Apparent molar mass kDa	ζ, mV	Z‐average size, nm	Surface tension mN m^−1^
HW‐H	813	183	5.5	−10.4 ± 0.9	316 ± 19	57.8
SW‐H	827	213	3.6	−24.3 ± 0.2	620 ± 10	47.3
HW‐L	527	414	4.3	−20.0 ± 0.5	2116 ± 30	56.6
SW‐L	117	842	1.7	−23.7 ± 0.2	1150 ± 55	40.4

Our results revealed a positive correlation between lignin content and particle size (Table 1), likely involving an association between the non‐cellulosic wood components. Finally, all the aqueous suspensions were anionic (zeta potential values between −10 and −25 mV), due to carboxylic groups in hemicelluloses and/or lignins, as well as galacturonic acids from pectin (Table 1).

**Figure 3:**
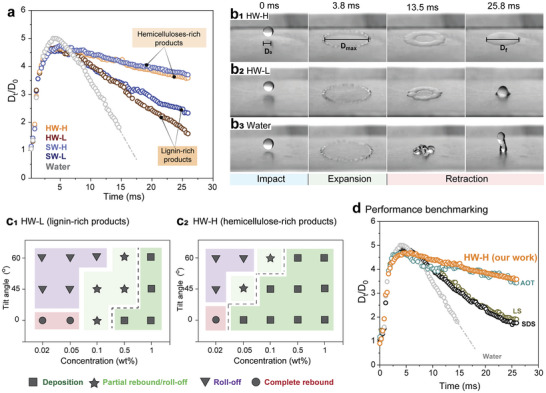
Droplet impact dynamics on model Parafilm surface. a) Evolution of droplet diameter during impact as a function of time for wood‐extracted fractions compared to water. The dotted line indicates droplet rebound from water. b) Droplet impact events corresponding to 0.1 wt.% hardwood hemicelluloses‐rich fraction (HW‐H), lignin‐rich fraction (HW‐L), and water on the Parafilm. c) Map of the droplet impact behavior of c_1_) HW‐L and c_2_) HW‐H as a function of concentration and tilting angle. d) Comparison of the HW‐H droplet diameter evolution compared with traditional surfactants used in the field, namely, sodium dodecyl sulfate (SDS), lignosulfonate (LS), sodium bis(2‐ethylhexyl) sulfosuccinate (Aerosol OT, AOT).

**Figure 4:**
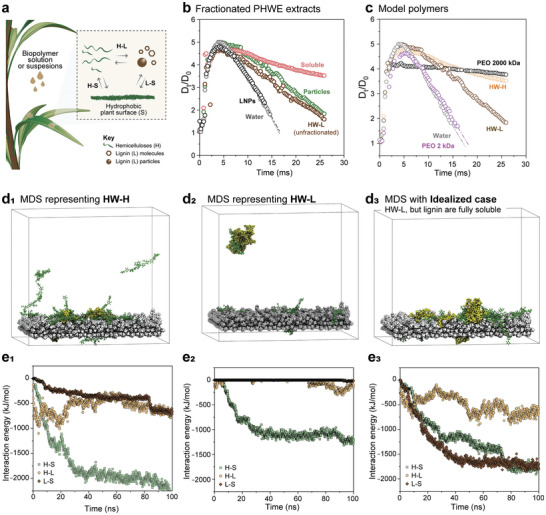
Rebound suppression mechanism of wood‐extracted fractions. a) Schematic of the biopolymer‐plant leaf interactions that support an understanding of the mechanism for droplet rebound control. b) Evolution of droplet diameter as a function of time for the soluble and particulate fractions obtained from hardwood lignin. c) Evolution of diameter as a function of time for droplets containing polyethylene oxide (PEO) (M_n_ 200 and 2 kDa) and HW‐L and HW‐H extracts. d) snapshots of the molecular dynamic simulation of systems representing d_1_) HW‐H, d_2_) HW‐L, and an d_3_) idealized case of HW‐L where the lignin components are small and soluble. e) Respective interaction energy profiles acquired for three main interactions taking place between hemicellulose, lignin, and plant surface (displayed in a) for the e_1_) HW‐H, e_2_) HW‐H, and e_3_) the given idealized case. Note: curves in e) have components from electrostatic interactions and Van der Waals forces, which are separated in Figures S14 and S15 (Supporting Information).

All samples comprised mixtures of lignin and hemicelluloses, with glucuronoxylans (GX) being the main hemicellulose type in the hardwood extracts. Meanwhile, galactoglucomannans (GGM) were predominant in the softwood fractions (Table S1, Supporting Information). We used HSQC NMR to qualitatively analyze HW‐L (soluble fraction in Figure S4, Supporting Information) and HW‐H. The aqueous suspensions of these specific samples displayed unique features, such as a clear presence of both soluble and colloidal fractions, which later were found to affect rebounding/adhesion (**Figures** **3** and **4**). Therefore, we used this sample for further fractionation and to deconvolute chemical and colloidal features on the droplet rebound suppression effect of our hemicellulose and lignin mixed extracts.

Acetyl groups (at ∼20;2.0 ppm) were identified in all samples (Figure S3, Supporting Information), indicating hemicellulose deacetylation and partial hydrolysis during the pressurized hot water treatment.^[^ ^21^ ^]^ Xylan was observed in HW‐L, both in colloidal aggregates (Figure 2e_1_) and in soluble state (Figure S4, Supporting Information). The same applied to HW‐H (Figure 2e_2_). The resonance intensity assigned to xylan in each of the analyzed samples followed closely the quantitative results obtained by composition analysis (Table S1, Supporting Information). Although very weak in HW‐H, the characteristic resonance of lignin was observed in all samples, displaying β–O‐4, β– β beta, and β–5 as the most common lignin interunit linkages. Most importantly, HSQC NMR indicated the presence of LCCs only in the lignin‐rich extract from hardwood. The most common LCC structures (Figure S4, Supporting Information) are phenyl glycoside (at ≈103–100; 5.2–4.7 ppm), benzyl ether 1 (at ≈80.2; 4.5 ppm), benzyl ether 2 (at ≈82; 5.2 ppm), and γ‐ester (at ≈64–62; 4.5–4 ppm).^[^ ^22^ ^]^ The particle fraction in HW‐L clearly showed the presence of benzyl ether 2 (BE2) structures, indicating LCC presence (Figure 2e_1_). Interestingly, this sample contained a high fraction of colloidal particles. We hypothesize that LCC either i) induced the assembly of lignin molecules into colloidal particles, or ii) formed precipitated particles with large lignin molecules of low solubility in water. This corroborates previous work on the assembly of LCC nanoparticles.^[^ ^23^ ^]^ The soluble HW‐L fraction displayed similar chemical features as those of the relatively purer hemicelluloses in HW‐H (Figure 2e_2_; Figures S3 and S4, Supporting Information).

Both hemicellulose‐rich and lignin‐rich fractions, at concentrations as low as 0.1 wt.%, reduced the surface tension of water to values below 60 mN m^−1^, reaching a minimum of 40 mN m^−1^ in the case of SW‐L (Table 1 and Figure S5a, Supporting Information). All the extracted fractions (released from birch and spruce by PHWE), displayed surface activity similar to that of lignosulfonates (Figure S5b, Supporting Information), which are commonly used in aqueous agrochemical formulations.^[^ ^24^ ^]^ Softwood‐derived fractions reached surface tension values similar to those of typical surfactants (at the same concentration), such as sodium bis(2‐ethylhexyl) sulfosuccinate [Aerosol OT (AOT)], sodium dodecyl sulfate (SDS) and trisiloxane surfactants (TS).^1^ Overall, the obtained lignin‐rich fractions were more surface‐active than their hemicellulose counterparts, whereas softwood‐derived components displayed the lowest surface tension.

The hydrophobic aromatic structure of lignin synergizes with hydrophilic groups of hemicellulose thus improving their colloidal mobility and promoting surface activity.^[^ ^25^ ^]^ This is the case of unmodified lignin‐rich fractions (HW‐L and SW‐L) that are composed mostly by lignin‐hemicellulose supramolecular complexes (Figure 2e). Supramolecular interactions between hemicelluloses and small residues of lignin favored the solubility of lignin at neutral pH, which can improve their efficiency as surface‐active compounds.^[^ ^26^ ^]^ Within our extracts, colloidal particles were not efficient in decreasing the surface tension of the biopolymeric mixtures. Simple lignin‐hemicellulose mixtures represent a viable solution to decrease surface tension of water, which has been previously exploited in the stabilization of oil‐in‐water emulsion.^[^ ^27^ ^]^

Lignin tends to self‐assemble into colloidal particles in the aqueous environment,^[^ ^28^ ^]^ thus decreasing the availability of surface‐active groups compared to its soluble counterpart. Therefore, the surface tension was drastically reduced when lignin was partially solubilized by increasing the pH of the HW‐L sample to 12 (Figure S6, Supporting Information). We observed that softwood's hemicellulose‐rich and lignin‐rich (SW‐H, SW‐L) fractions were more surface‐active than the hardwood counterparts (HW‐H, HW‐L). The wood components released from softwood during the aqueous extraction were smaller (Table 1), favoring their diffusion and adsorption at air‐water interfaces. However, reduced surface tension is only one of the factors playing a role in droplet impact control and it is not necessarily directly correlated, as we demonstrate next.

### 2.2 Droplet Impact on a Model Parafilm Surface

Although softwood‐derived fractions showed lower surface tension (Figure S5, Supporting Information), they performed similarly in bouncing tests compared to those obtained from hardwood. Significant differences, however, were observed considering the fractions rich in lignin or hemicelluloses (Figure 3a). To study the effects on droplet rebound from hydrophobic surfaces, we next discuss the HW‐L and HW‐H fractions, with a focus on the hemicellulose and lignin in the soluble or colloidal states. Solutions/suspensions of all extracted products displayed viscosity similar to water (0.85–0.88 mPa.s) at the highest concentration investigated (1 wt.%), therefore stating a neglectable effect of viscosity on the water droplet impact behavior within the studied biomolecules and their concentration range.

A Parafilm film (water contact angle, WCA = 110°) was used as a model hydrophobic surface. Water droplets containing or not wood adjuvants underwent i) impact, ii) spreading, and iii) retraction (Figure 3b), following deposition or rebound from the hydrophobic surface. Figure 3a shows the evolution of the droplet diameter (D_t_/D_0_) (case of HW‐H and HW‐L at 0.1 wt.% concentration), as well as pure water, used as reference (see also photos taken with a high‐speed camera in Figure 3b_1_–b_3_). The initial impact and expansion stages were governed by inertial forces. Droplets (≈3 mm) were split at high velocity causing inertial‐dominated impact and expansion. All droplets (except pure water) expanded to reach a maximum diameter at ≈3.8 ms after impact. This was followed by a retraction phase, during which viscous and capillary forces (driven by surface tension) governed wetting and adhesion.^[^ ^4^ ^]^ The high surface tension of water contracted the droplet and accelerated the retraction.

Lignin‐rich extracts partially rebounded while droplets of hemicelluloses‐rich extracts did not rebound. None of the droplets showed breakage nor splashing during any of the phases (impact, expansion, and retraction), which allowed us to assess the evolution of droplet diameter (D_t_ /D_0_) and retraction speed. Figure 3a indicates that the final droplet diameter of hemicelluloses‐rich extracts (from both hardwoods and softwoods) was higher than that of lignin‐rich extracts (as well as water). Moreover, the retraction speed of the impacting droplets (Figure S7, Supporting Information), calculated from the profiles in Figure 3a, confirmed the superiority of wood‐based solutions/suspensions obtained by PHWE to adhere to hydrophobic surfaces when compared to water, and that PHWE hemicelluloses were more effective than their lignin‐rich counterpart. Note that the extracts are not pure in one or the other wood biopolymer, but rather rich in hemicellulose or lignin. The effect of each individual component will be decoupled next, using reference samples, and fractionated extracts.

When mapping the effect of HW‐L (lignin‐rich) and HW‐H (hemicellulose‐rich) as far as concentration of the solutions/suspensions and tilt angle of the target surface (Figure 3c_1_,c_2_), we observed that HW‐H promoted adhesion on flat substrates, even at concentrations as low as 0.05 wt.%. On surfaces tilted at a 65° angle, a higher concentration of the hemicellulose fractions was needed, but the adhesion still took place in a dilute regime (0.5 wt.%). Therefore, droplet rebound was completely inhibited in hemicellulose‐rich fractions, an effect that was comparable to AOT (a typical surfactant used in the field, Figure 3d; Figure S8, Supporting Information). Remarkably, the PHWE wood‐extracted fractions were more efficient than SDS and lignosulphonate (LS) solutions used at the same concentration (Figure 3d). Droplet roll‐off was suppressed from tilted hydrophobic surfaces for HW‐H from 0.1 and 0.5 wt.% concentration (Figure 3c_2_). A minimum concentration of 1 wt.% was required for HW‐L to achieve the same performance (Figure 3c_1_).

The hemicellulose‐rich fractions were mostly solubilized in water while lignin‐rich fractions were mostly present as particles. The hemicellulose‐rich fractions contained residual amounts of lignin (Table 1). The mechanism behind the improvement of droplet adhesion by rebound suppression is a result of a complex balance of interactions between hemicellulose, lignin, and the plant surface (Figure 4a). In order to further understand the effect of biopolymer‐surface interactions we proceeded using i) fractionated samples as well as model particles and polymers, and ii) molecular dynamic simulations of model systems mimicking our extracts and complementary idealized model cases.

First, we performed size fractionation of the HW‐L to compare the performance of PHWE wood‐extracted compounds against nanoparticles and polymers. HW‐L sample was chosen since it has enough composition variability (both chemical and colloidal/soluble) to promote various fractions that can help us understand and decouple the effect of soluble and colloidal fractions. In turn, this was found useful to further understand the underlying mechanism of droplet bouncing. As far as droplet rebound, HW‐L suspensions outperformed reference lignin nanoparticles (LNPs) externally prepared by the solvent shifting process^[^ ^29^ ^]^ (the LNPs behaved similarly to pure water) (Figure 4b). Colloidal micro‐ and submicron particles account for ca. 80 wt.% of HW‐L (determined by size fractionation, Figure S1, Supporting Information) with the remaining composed of soluble molecules, either residual lignin or hemicelluloses. To decouple the role of soluble molecules and particles and to gain further insights into the role of soluble/colloidal interactions on retraction, we investigated the dynamics of droplet impact considering fractionated colloidal micro‐and nanoparticles and soluble molecules from HW‐L (Figure 4b) as well as their mixtures (1:1 ratio) (Figure S9, Supporting Information).

After stepwise centrifugation‐resuspension cycles, we obtained a translucent lignin fraction (ca. 50 nm measured by DLS), which concentrated the initially present soluble lignin and lignin‐hemicellulose complexes. We then tested the droplet impact behavior of these fractions following the same experimental procedures. Figure 4b shows the impact and retraction of 0.1 wt.% fractionated colloidal microparticles (2.5 µm) and the soluble part of HW‐L. Droplets with microparticles showed partial rebounding during the retraction, similar to the unfractionated HW‐L. Meanwhile, the soluble fractionation (at 0.1 wt.%) slowed down droplet retraction (Figure 4b), similar to hemicellulose‐rich fractions (Figure 3a). This is a result solely attributed to surface pinning since the soluble and particle‐rich fractions displayed similar surface tension (Figure S10, Supporting Information). The effect of particle size on retraction was not significant, as tested for particles sizes of ca. 2, 1.2, and 0.7 µm (visual observation).

The similar D_t_/D_0_ profiles of unfractionated and particle‐rich HW‐L (Figure 4b) revealed that the contribution to adhesion by the soluble fraction (ca. 20 wt.%) in unfractionated HW‐L was minimal. No synergistic effect was noted as far as the retraction speed for mixtures (1:1 ratio) of colloidal particles and the soluble fraction. In fact, the mixture behaved similarly as the prepared lignin colloidal particles (Figure S9, Supporting Information). Therefore, it is reasonable to conclude, from an experimental point of view, that a threshold concentration of soluble components is required to induce droplet adhesion on hydrophobic surfaces. For instance, the retraction speed decreased significantly (from 0.2 to 0.1 ms^−1^) in the case of 1:1 particle:soluble fraction mixture at a total solid content of 0.2 wt.% (even in the presence of the same ratios of colloidal particles, Figure S9, Supporting Information). At this concentration, the HW‐L soluble fraction could even reduce the rebound of aqueous suspensions of SiO_2_ microparticles (1.15 µm diameter), which are chemically inert and behave similarly to water as far as the droplet impact tests (Figure S11, Supporting Information).

The unfractionated HW‐H sample comprised mostly soluble compounds, with ca. 80 wt.% being oligomeric carbohydrates (ca. 5 kDa). Such fraction was only partially surface‐active, but performed slightly better than the reference surfactant, AOT. Therefore, to gain further experimental insights into the mechanism of rebounding, we used low and high‐molecular weight polyethylene oxide (PEO) as a reference compound for the HW‐H and HW‐L fractions (Figure 4c). PEO is used as a wetting adjuvant that acts by a process different than surface activity.^[^ ^4^ ^]^ This is on the basis that long‐chain molecules extend (by shear forces) while the droplet spreads to a given degree on the target surface, creating local pinning on the surface, and resisting the droplet retraction. This is typically observed for high molecular weight polymers (M_n_ of 2000–4000 kDa) where long chains undergo stretching upon lateral deformation during the droplet impact. However, the retraction performance of non‐surface active HW‐H fractions is unique considering their oligomeric size. As far as droplet rebound, Figure 4c shows that HW‐H performed similarly to PEO (M_n_ 2000 kDa) and outperformed oligomeric (M_n_ 2 kDa) and low molecular weight PEO (M_n_ of 100 kDa, Figure S12, Supporting Information). The PEO (M_n_ of 2 and 100 kDa) solutions rebounded like water, whereas HW‐H (M_n_ of ca. 5 kDa) greatly reduced droplet rebound.

Not only the content but also the nature of the lignin components factors for the performance of the wood extract on controlling droplet rebound. In HW‐H, lignin is encountered mostly as lower molecular weight molecules, which promote better adhesion than their colloidal counterparts. Molecular dynamic simulations (MDS) were employed to assess the interactions taking between hemicellulose, lignin, and substrate during droplet impact. We note that there is a difference between the time scale of the experiments (milliseconds) and simulations (pico‐nanoseconds); however, the idealized, modeled cases help us to understand the generic interactions that are affecting the binding performance of the biopolymers toward hydrophobic surface. We modeled the total interaction energy, and its components related to electrostatic interactions and Van der Waals forces for hemicellulose‐substrate (H‐S), lignin‐substrate (L‐S), and hemicellulose‐lignin (H‐L) paired systems (Figure 4a). A representative birchwood hemicellulose featuring a xylose‐to‐4‐O‐methylglucuronic acid ratio of ≈10:1 was used as a model compound,^[^ ^30^ ^]^ while two lignin structures with molecular weight of ≈1.6 and ≈7.2 kDa, with syringyl:guaiacyl ratio equal to 0.99:1,^[^ ^31^ ^]^ were constructed and used as lignin soluble and colloidal entities. Polyethylene was used as a model hydrophobic surface. Snapshots of the simulations are represented in Figure 4d_1_–d_3_ representing the samples HW‐H, HW‐L, and an idealized case where HW‐L has the same lignin content but in fully soluble state (i.e., smaller molecules).

In the HW‐H modeled system, lignin and hemicellulose interact stronger at the early stages (Figure 4e_1_) via electrostatic interactions (Figure S14a, Supporting Information), and then the interaction energy decays over time. Both lignin and hemicellulose proportionally contribute to the overall interaction energy toward the substrate, but hemicellulose displays more pronounced interactions with the substrate taking place via Van der Waals forces (Figure S14a, Supporting Information). The interaction energy reaches a plateau at very short time span, which is likely due to the H‐L hydrogen bonding favoring the H‐S and L‐S VdW contact interactions with the substrate. Bigger lignin colloidal components, like those observed in HW‐L, reduce drastically all the interactions between hemicellulose‐lignin‐substrate. In fact, in this system the only component contributing to the adhesion, and consequently to preventing droplet rebound, are hemicelluloses. Colloidal lignin, minimizes all electrostatic interactions taking place between the three components in the system, thus relying only on H‐S VdW forces for suppressing the rebounding (Figure 4e_2_; Figure S14b, Supporting Information). This is confirmed by the behavior of lignin colloids during droplet impact experiments, which were unable to create strong local pining due to weak interactions with the surface, and ultimately causing higher retraction speed and partial droplet rebound (Figure 4b; Figure S11, Supporting Information). However, we note that lignin can also induce high adhesion (and rebound control), provided that they are in soluble state (Figure S11, Supporting Information). To confirm our experimental observations, we created an idealized case in which the lignin content in HW‐L (with original hemicellulose:lignin ≈1:1, and lignin mostly in colloidal state) is fully soluble (Figure 4e3; Figure S14c, Supporting Information). In this scenario, L‐S and H‐S are equally strong, both driven by Van der Waals forces (Figure S14c, Supporting Information), while simultaneously strongly interacting with each other (H‐L) via electrostatic interactions, i.e., hydrogen bonding, during the whole simulation. Nevertheless, pure hemicellulose solutions, under simulation, display higher interactions energy than that of hemicellulose‐lignin mixture (Figure S15, Supporting Information). However, pure hemicellulose solutions require additional purification steps for removing persistent residual lignin entities, thus decreasing the feasibility of the potential product or technology. This may impede their utilization in large‐scale and low‐cost applications such as those in agriculture.

Based on our experimental observations and modeling results, we can confirm that the presence of small and soluble lignin molecules along with hemicelluloses favored Van der Waals contact interactions between the biopolymers and the surface, which takes places while the droplet stretches perpendicularly to the impact direction. Shear forces extend hemicellulose polymers laterally while pushing the more “3D” lignin molecules toward the surface. The highly adhesive polydentate nature of low molecular weight plant polyphenols^[^ ^32^ ^]^ enables the creation of “bridging” electrostatic interactions (hydrogen bonding) with the hemicelluloses, thus promoting spreading and retention of the droplets on the surface via VdW forces (Figure 4d). It is known that lignin is an effective adhesive in the soluble state, and that lignin powders^[^ ^33^ ^]^ and particles^[^ ^34^ ^]^ can improve the performance of adhesives. However, here, pinning and adhesion phenomena during droplet impact takes place in a timescale of the order of milliseconds; therefore, lignin must display high mobility to move from the droplet bulk to the solid‐liquid interface. Our droplet impact experiments, and modeling system clearly reflected the importance of solubility for an effective suppression of droplet rebound by using wood biopolymers.

### 2.3 Dynamics of Droplet Impact on Citrus and Cauliflower Leaves

**Figure 5:**
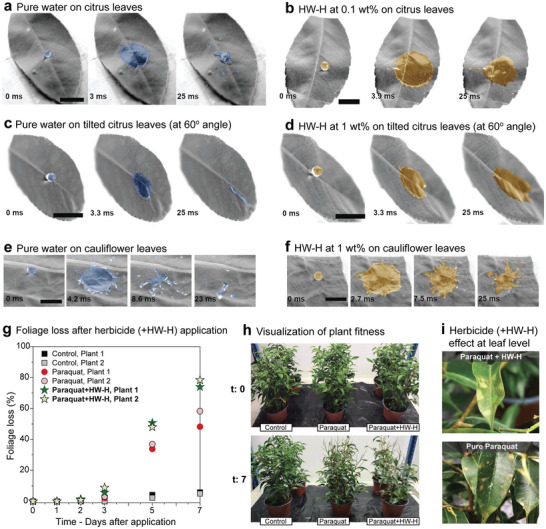
Droplet impact and adhesion on plant leaves. Time‐lapsed droplet impact on flat citrus leaves considering a) pure water and b) 0.1% aqueous solution of hardwood hemicelluloses‐rich fraction (HW‐H). Impact dynamics on 60° tilted citrus leaves shown by droplets of c) pure water and d) 1 wt.% HW‐H. Impact dynamics on flat cauliflower leaves are shown for e) pure water and f) 1 wt.% HW‐H droplets. Scalebars are 1 cm. g) Foliage loss calculated, as a percentage of leaves, along the first seven days after the herbicide formulations were applied. h) Photographs displaying the aspect of the plant right after the application (t: 0, top), and after seven days (t: 7 days, bottom). i) Visual aspect of the herbicide action at a leaf level for herbicide added of 1 wt.% of HW‐H (top), and pure herbicide (bottom). Note: in images (a–f) the droplets were artificially colored in a software (i.e., no dye was utilized during the droplet impact experiment).

After understanding the mechanisms of rebound suppression by hemicellulose and lignin mixtures, we tested our extracts in freshly harvested plant leaves. Droplet retention on foliage surfaces is challenged by their waxy nature featuring hairy, curved, or rough structures. Microstructured surfaces induce asymmetric droplet impact, leading to an increased droplet splitting and low deposition.^[^ ^6^ ^]^ Herein, we investigated droplet impact on citrus (WCA = 95°) and cauliflower (WCA = 145°) leaves. We followed the impact of water drops with and without PHWE hardwood hemicellulose‐rich fractions (HW‐H) on flat and 60° titled citrus leaves (**Figure** **5**). We chose HW‐H since it was the best performer during the measurement in the model surfaces. On flat citrus leaves, pure water drops were spread, and receded while breaking, as observed during the retraction (Figure 5a). In contrast, HW‐H drops (0.1 wt.%) spread over a larger area, with minimum break up during retraction (Figure 5b). The effect of HW‐H was pronounced on 60° titled citrus leaves since drop roll‐off from leaf surface was inhibited (results in Figure 5c compared to Figure 5d); however, the concentration required for HW‐H to achieve such effect was 1 wt.%. On the more hydrophobic cauliflower leaves, pure water droplets started to break soon after impact (Figure 5e). Figure 5e shows droplet splashing and rebounding during retraction, after 4.2 ms. This was prevented, at least to some extent, when using HW‐H (1 wt.%, Figure 5f); however, complete deposition was not observed (partial roll‐off occurred on titled cauliflower leaves). The performance of HW‐H extracts in controlling droplet rebound from citrus leaves was comparable to that of model surfaces.

The latter results highlight the challenges imposed by adhesion on textured or curved surfaces. Surfactants and polymer additives are used to increase the contact time, harness the surface tension reduction and pinning capacity.^[^ ^6^ ^]^ HW‐H adjuvant improved retention on commercially relevant plant leaves by a factor of 10. This can decrease for example herbicide run‐off or improve nutrient application. In summary, fully biobased HW‐H should not impose any environmental risk given its non‐toxicity and biodegradation, and it performs at a similar level compared to surfactant‐based formulations.

Next, we assess the effect of adding 0.1 wt.% of HW‐H on the efficiency of a paraquat (herbicide) solution. Paraquat is a known contact herbicide that acts by disrupting photosynthesis and rupturing plant cell membranes, allowing water to escape from plant tissues and leading to their dissection.^[^ ^35^ ^]^ Although some plants are more resistant to fully dying from the effect of the herbicide, foliage loss is ubiquitously seen across all higher plants. In our study, we used negative (only water) and positive (pure paraquat at same concentration) controls to investigate a new formulation containing both paraquat and the HW‐H. Plants were sprayed with a respective solution, followed by frequent water mist in the subsequent days to simulate dew and associated herbicide run off, while their foliage loss was determined (Figure 5g). Plants sprayed with the herbicide positive control and herbicide‐adjuvant formulation demonstrated initial foliage loss at day 3 after application, which increased constantly until reaching ca. 80% foliage loss for the paraquat/HW‐H formulation compared to ca. 60% for pure paraquat. This highlights that the HW‐H extract not only reduces water droplet bouncing but also significantly decreases herbicide run off thus improving its overall efficiency. Visually, all plants exposed to herbicide significantly lost their foliage and changed coloration into more pale shades of green – due to dissecation (Figure 5h). Figure 5i demonstrates the similar effects of the herbicide at a plant leaf level, regardless of the presence of the adjuvant. The leaf extracted from the plant treated with Paraquat added of HW‐H, however, shows bigger yellowish areas, which comes from a better spread of the formulation droplets on the leaf surface. This is supported by UV‐Vis spectra of pesticide‐adjuvant mixtures (Figure S16a, Supporting Information), which do not show any strong interactions between them, although the zeta potential of the HW‐H slightly changes thus potentially leading to a slightly less stable formulations (Figure S16b, Supporting Information).

## 3 Conclusion

Aqueous fractions isolated by pressurized hot‐water flow‐through extraction (PHWE) of wood are enriched in hemicelluloses and lignin of low molecular weight. They are only partially surface‐active but compete or outperform synthetic agents used to minimize droplet rebound from hydrophobic surfaces. The effect of wood‐based extracts is a result of molecular extension (hemicelluloses) combined with highly adhesive lignin with the hydrophobic surface via secondary interactions. PHWE hemicelluloses‐rich extracts are effective to prevent droplet rebound compared to lignin‐rich fractions on Parafilm surface (0.05, 0.1, and 0.5 wt.% on flat, 45, and 60° tilted surfaces, respectively). Soluble molecules contained in hemicelluloses‐rich fractions expand during droplet expansion and pins the surface, slowing down droplet retraction. This pinning is not observed with lignin‐rich extracts composed of microparticles. This is not seen in the case of PEO with an oligomeric size like that of hemicelluloses, supporting the role of lignin and/or lignin‐derived moieties in the adhesion process. Lignin residues in hemicellulose‐rich extract support the pinning mechanism by promoting adhesion between hemicelluloses and the hydrophobic leaves via supramolecular interactions, taking advantage of their polydentate polyphenol character. Hemicellulose‐rich fractions prevent roll‐off on citrus leaves and improve leaf coverage by a factor of 10. Our hemicellulose‐lignin adjuvant was able to better retain herbicide molecules on model plants, thus increasing its overall pesticide efficiency. Our findings pave the way to the utilization of non‐cellulosic components as biobased wetting adjuvants to minimize the environmental impacts of agrochemical pollution from chemical surfactants. Additional benefits can be expected as far as UV protection and bioactivity of lignin and foliar delivery of agrochemicals.

## 4 Experimental Section

### Materials

Dioctyl sulfosuccinate sodium salt (AOT) and sodium dodecyl sulfate (SDS) were sourced from Merck (Darmstadt, Germany). Paraquat dichloride hydrate (CAS 75365‐73‐0), nonionic polyethylene oxide (PEO, number‐average molecular weight M*_n_* 2 kDa and 200 kDa) were supplied by Merck (Darmstadt, Germany). Silica particles (1.15 ± 0.03 µm) were from Microparticles GmbH (Lot no. SiO_2_‐R‐L3705, Berlin, Germany). Lignosulfonates were from Domsjö, Sweden. Parafilm was purchased from Merck (Darmstadt, Germany). Citrus leaves were obtained from dwarf citrus plants bought from Plantagen Finland. The variety was a cross between mandarin and kumquat. Fresh citrus leaves were plucked before the measurement from the citrus plant grown at normal room temperature. Cauliflower leaves were bought fresh from a local supermarket. All leaves were washed with pure water to remove dust/dirt, air‐flushed with pressurized air to remove water, and kept at room temperature for 1–2 h before the experiments.

### Pressurized Hot Water Extraction

Different routes, summarized in Figure 2a, were applied to produce lignin and hemicellulose‐rich fractions from birch and spruce. Sawdust from hardwood birch and softwood spruce was subjected to pressurized hot water extraction in a flow‐through system according to Kilpeläinen et al.^[^ ^36^ ^]^ To obtain a lignin‐rich fraction, sawdust was extracted at 170 °C for 60 min using 20 kg min^−1^ flow‐through condition. The lignin‐rich fraction was separated by precipitation of the intermediate bulk container (IBC) that collected the flow‐through extracts for 20–60 min. Only the birch sawdust was pre‐steamed at 125 °C before the flow‐through extraction. These fractions were obtained as powders and used as such.

Physical separation of soluble and colloidal portions of the lignin‐rich fraction was performed by centrifugation of a 1 wt.% suspension of lignin‐rich fraction obtained from birch using a Beckman Coulter Optima L‐90K centrifuge (Beckman Coulter, California, USA) operated with a fixed angle 70 Ti rotor. A particle‐rich fraction was obtained as a precipitate after centrifugation (1840 × *g*, 5 min). The supernatant was further centrifuged (66 200 × *g*, 10 min) to obtain the soluble fraction. A mass balance of these fractions was performed gravimetrically.

To obtain hemicelluloses‐rich fractions from birch, sawdust was extracted at 180 °C for 50 min with 13 kg min^−1^ flow followed by ethanol precipitation of the immediate hydrolysate with 1/10 extract/ethanol (vol/vol). The precipitate was filtered and vacuum‐dried at 40 °C to obtain a powder. A hemicelluloses‐rich fraction from spruce was obtained from pre‐steamed sawdust at 114 °C, followed by flow‐through extraction with 20 kg min^−1^ at 170 °C for 60 min. The hydrolysate was concentrated with tubular 6‐kDa PCI‐membranes modified polyethersulphone after pH adjustment to 9.8, concentrated, and spray dried with Mobile Minor spray dryer (GEA Niro A/S, Denmark).

### Carbohydrate and Lignin Analyses

Non‐cellulosic carbohydrates in the samples were quantified as sugars using a gas chromatography‐flame ionization detector after acid‐methanolysis treatment following Sundheq et al.^[^ ^37^ ^]^ Total carbohydrates were calculated from the sum of neutral and acidic monosaccharides in birch and spruce hemicelluloses (arabinose, xylose, rhamnose, mannose, galactose, glucose, 4‐O‐methyl glucuronic acid, galacturonic acid). Total lignin content was measured by adding the acid‐soluble and insoluble lignin in 72% sulfuric acid according to Sluiter et al.^[^ ^38^ ^]^

### Molar Mass Analysis

The molar mass of the samples was determined with an Agilent 1260 Infinity II Multi‐Detector size‐exclusion chromatography unit equipped with light‐scattering (15° and 90°), refractive index, differential viscometer (VISC), and UV detectors. Agilent PLgel MIXED‐B 7.8 × 300 mm columns in dimethyl sulfoxide (DMSO) were used at a flow rate of 0.7 mL min^−1^. Samples were solubilized in DMSO for 2–3 days and injected at a concentration of 1–2 mg mL^−1^ in dimethyl sulfoxide (DMSO). No visible sediment was observed in DMSO. The injection volume was 100 µL. The columns were calibrated using narrow dispersity pullulan standards. The molar mass was estimated from the pullulan calibration curve acquired by injecting seven standard pullulans with molar mass ranging from 100 to 110 000 g mol^−1^.

### Multiplicity‐Edited HSQC

The HSQC experiments on wood extracted fractions were done using a multiplicity‐edited phase‐sensitive HSQC sequence with a sensitivity‐improved multiplicity‐edited phase‐sensitive HSQC sequence with echo/antiecho‐TPPI gradient selection and adiabatic pulses (Bruker pulse program ‘hsqcedetgpsisp2.2′), for increased sensitivity.^[^ ^39^ ^]^ Parameters are as follows: spectral widths (sw) were 20.00 and 220.00 ppm, with transmitter offsets (o1p) of 5.00 and 100.00 ppm, for ^1^H and ^13^C dimensions, respectively. The time‐domain size (td1) in the indirectly detected ^13^C‐dimension (f1) was 256, corresponding to 128 increments, for the real spectrum. There were 128 dummy scans (ds), typically 192 scans (ns), an acquisition time (aq) of 0.205 s for f2, and a relaxation delay of 1.0 s. Window functions were typically sine squared (90 o) in f1 and f2.

### Particle Size and Zeta‐Potential Analyses

The particle size and zeta‐potential analyses of extracts in water (pH 6) were performed using a Zetasizer Nano ZS 90 (Malvern Instruments, Worcestershire, UK) at 22 °C. Z‐average of particle size and zeta (ζ)‐potential are presented as mean and standard deviation from at least three measurements.

### Scanning Electron Microscopy (SEM)

Images were acquired using field‐emission SEM Sigma VP (Zeiss Instruments, Jena, Germany). Silicon wafers were cleaned with water and ethanol before plasma cleaning. Afterward, the wafers were dip‐coated with dilute suspensions and dried overnight at room temperature. Before imaging, a 4 nm thick layer of gold or iridium was sputtered. Imaging was performed in a vacuum at an accelerating voltage of 1.5 kV.

### Surface Tension and Contact Angle Measurement

The surface tension was measured using the Theta Optical Tensiometer (Biolin Scientific) using the pendant drop method. The surface tension values of a 10‐µL suspension were recorded against air for 10 000 s at 0.14 fps. Contact angles (sessile drop method) were measured using an Attention Theta Optical Tensiometer equipped with an automated liquid pumping system. A 5–6 µL droplet was placed on the hydrophobic substrate (Parafilm or leaves) and the contact angle was measured for 15 s after the droplet was placed on the surface for 1 s.

### Droplet Impact

A 20‐µL drop of the given samples was released (pipetted) at a height of 55 cm above the solid substrate. The impact event was recorded using a high‐speed camera (Phantom Miro LC310, Vision Research Inc., USA) at 3631 frames per second (fps) with a viewing angle of 65° or 30°. Recorded videos were analyzed to monitor the evolution of droplet diameter, reported as D_t_/D_0_ as a function of time, where D_0_ is the initial diameter. The retraction speed was obtained from D_t_/D_0_ versus the time (*t*) curve.

### Molecular Dynamic Simulation

The hemicellulose strand derived from birchwood samples was constructed with a xylose‐to‐4‐O‐methylglucuronic acid ratio of ≈10:1,^[^ ^30^ ^]^ which was partially O‐acetylated, with a degree of acetylation of ≈0.2.^[^ ^40^ ^]^ This hemicellulose structure consists of 11 sugar units, with a molecular weight of 1738.51 Da. Among these, 2 xylose units were O‐acetylated (Figure S13, Supporting Information). For the lignin molecules, two types of lignin with the molecular weight of 1643.71 and 7225.48 Da were used. The weight ratio of syringyl‐to‐guaiacyl equal 0.99:1.^[^ ^31^ ^]^ The complex structures studied in this research, comprising hemicellulose and lignin, were constructed with hemicellulose‐to‐lignin weight ratios of 4.23:1, 1.05:1, and 1.1:1 (for large lignin molecules), as well as pure hemicellulose systems. All these structures were built using the Packmol package.^[^ ^41^ ^]^

The topological files of hemicellulose and lignin were generated using Charmm‐Gui glycan modeler and lignin builder, respectively.^[^ ^41^, ^42^ ^]^ All the atomistic simulations described herein were conducted by using NAMD,^[^ ^43^ ^]^ version 3.0, patched with the latest version of Colvars.^[^ ^44^ ^]^ In all simulations, CHARMM36 Force Field was used for hemicellulose, lignin molecules, substrate (polyethylene), and TIP3P water model.^[^ ^45^ ^]^

A preliminary equilibrium simulation was performed in the *N.P.T*. ensemble at a temperature of 298.15 K and a pressure of 1 bar controlled by Langevin dynamics^[^ ^46^ ^]^ and Langevin piston method,^[^ ^47^ ^]^ respectively. The simulation was carried out for a duration of 1 nanosecond to ensure that the solvent molecules reached a state of equilibrium and to eliminate unreasonable overlaps of neighboring atoms. Subsequently, the production simulation trajectories were advanced in the *N.P.T*. ensemble for a period of 100 ns. The particle mesh Ewald (PME) method was used in all simulations to compute long‐range electrostatic interactions. A spherical cutoff of 12 Å was employed to truncate short‐range Van der Waals (VdW) and electrostatic interactions. The equilibrium length of the bonds between each hydrogen and its respective mother atoms was maintained by applying SHAKE/RATTLE algorithms.^[^ ^48^ ^]^ The intermolecular (non‐covalent) interactions were analyzed using simulation trajectories generated with NAMD. The topology files were converted to the GROMACS format using VMD TopoTools (TopoGromacs),^[^ ^49^ ^]^ and the analysis was conducted with the GROMACS gmx energy utility.^[^ ^50^ ^]^

### Proof‐of‐Concept Testing of Herbicide Formulations Containing Wood‐Based Adjuvants

The efficiency of a selected wood‐based adjuvant (HW‐H) to improve the retention of biochemicals on the plant foliage was tested using a common contact herbicide, namely Paraquat. For these experiments *Ficus benjamina* plants, freshly purchased from a local store (Bauhaus – Finland) were used. Each plant was labeled and had its leaves counted. Two plants were sprayed only with water, while two were sprayed with pure paraquat solutions at 1 g L^−1^ concentration, with the remaining two being sprayed with paraquat added of HW‐H, both at 1 g L^−1^ concentration each. A total active component application of 2 kg ha^−1^ was aimed for, a common recommendation for Paraquat herbicide.^[^ ^51^ ^]^ Each plant was individually sprayed with the respective formulation and let to dry overnight. During the subsequent days (from day 1 to day 7), each plant had an even water mist applied to simulate dew, and the eventual herbicide runoff that was associated to it. The number of lost leaves was obtained daily, and a foliage percentage loss was calculated based on this visual observation. Nevertheless, UV–vis spectra and zeta potential measurements of the herbicide/HW‐H solutions were recorded.

### Statistical Analysis

Descriptive statistical analysis was used to describe the data obtained in the experiments related to the droplet impact results. All the results were average of at least three replicates. The normal distribution of the data was verified before plotting the averaged result along with its standard deviation. New experiments were performed whenever outliers were identified. Qualitative chemical analysis (e.g., HQSC NMR) as well as imaging techniques were done using mixed triplicates of the extracts, with random sampling in order to ensure a representative sample. All data treatment, including plotting, was done using Origin 2025.

## Conflict of Interest

The authors declare no conflict of interest.

## Supporting information



Supporting Information

## Data Availability

The data that support the findings of this study are available from the corresponding author upon reasonable request.
