# Supplementary material for: Wood Biomolecules as Agricultural Adjuvants for Effective Suppression of Droplet Rebound from Plant Foliage
Source: Adv Sci (Weinh). 2025 Mar 10;12(17):2416686. doi: 10.1002/advs.202416686 (PMC12061315; doi:10.1002/advs.202416686)
Supplement: Supplementary file 1 — Supporting Information [file ADVS-12-2416686-s001.docx]

Supporting Information

**Wood biomolecules as agricultural adjuvants for effective suppression of droplet rebound from plant foliage.**

*Mamata Bhattarai^*^, Hedar Al-Terke, Kai Liu, Zhangmin Wan, Petri Kilpeläinen, Alistair W. T. King, Alexey Khakalo, Jiayun Xu, Chunlin Xu, Robin H. A. Ras, Bruno D. Mattos^*^, Orlando J. Rojas^*^*

M. Bhattarai, B. D. Mattos, O. J. Rojas

Department of Bioproducts and Biosystems, School of Chemical Engineering, Aalto University, FI-00076, Espoo, Finland

E-mail: [mtabhattarai@gmail.com](mailto:mtabhattarai@gmail.com), [bruno.mattos@aalto.fi](mailto:bruno.mattos@aalto.fi), [orlando.rojas@aalto.fi](mailto:orlando.rojas@aalto.fi)

H. Al-Terke, K. Liu, R. H. A. Ras,

Department of Applied Physics, Aalto University, FI-00076 Espoo, Finland

Centre of Excellence in Life-Inspired Hybrid Materials (LIBER), Aalto University, Espoo, Finland

P. Kilpeläinen

Production Systems | Biomass fractionation technologies, Natural Resource Institute Finland (LUKE), Viikinkaari 9, 00790 Helsinki, Finland

A. W. T. King, A. Khakalo

Bioinspired Materials | Cellulose Coatings and Films, VTT Technical Research Centre of Finland Ltd., Tietotie 4E, FI-02044 Espoo, Finland

J. Xu, C. Xu

Laboratory of Natural Materials Technology, Åbo Akademi University, Henrikinkatu 2, FI-20500 Turku, Finland

Z. Wan, O. J. Rojas

Bioproducts Institute, Department of Chemical & Biological Engineering | Department of Chemistry | Department of Wood Science, The University of British Columbia, Vancouver, BC Canada V6T 1Z3

E-mail: [orlando.rojas@ubc.ca](mailto:orlando.rojas@ubc.ca)

**This file contains 16 figures and 2 tables in 19 pages.**


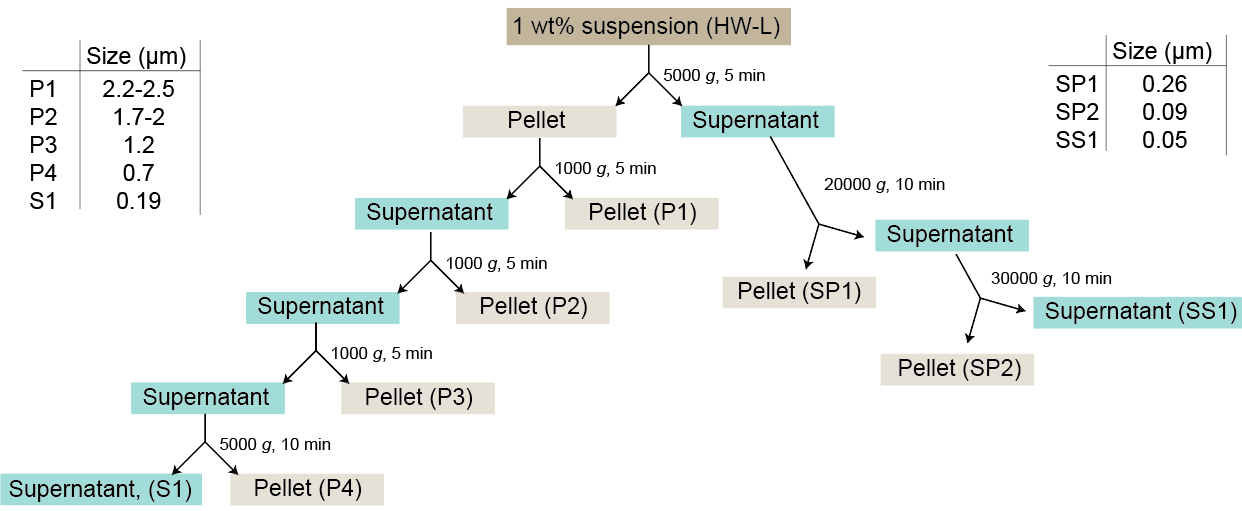


**Figure S1**. Centrifugation process developed to size-separate lignin-rich products.


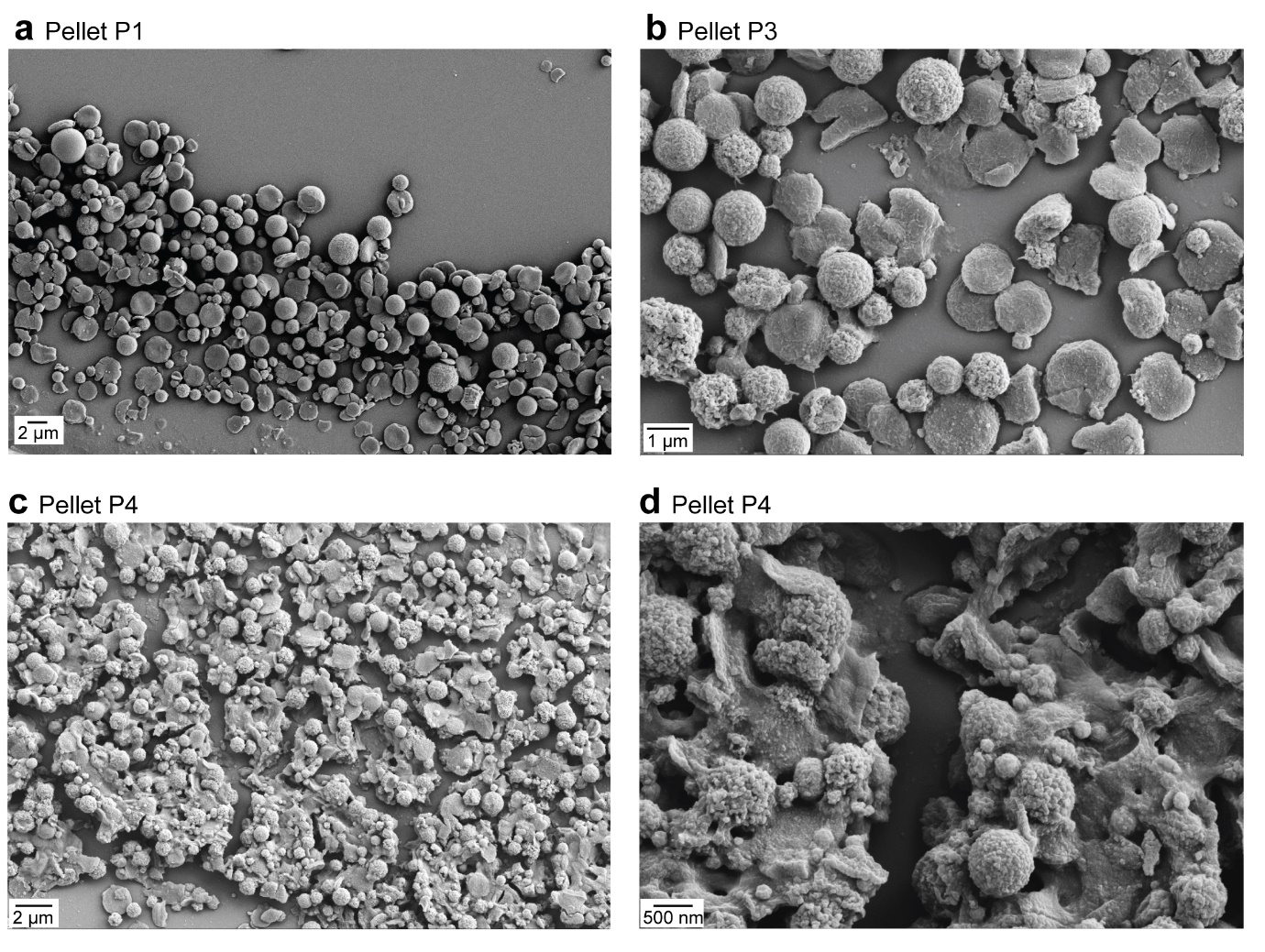


**Figure S2**. Scanning electron microscopy images of fractions obtained from lignin-rich products, mostly composed by **a-b**) particles and a mixture of **c-d**) particles and soluble. Fractions P1 to P4 are obtained according to the scheme in Figure S1.


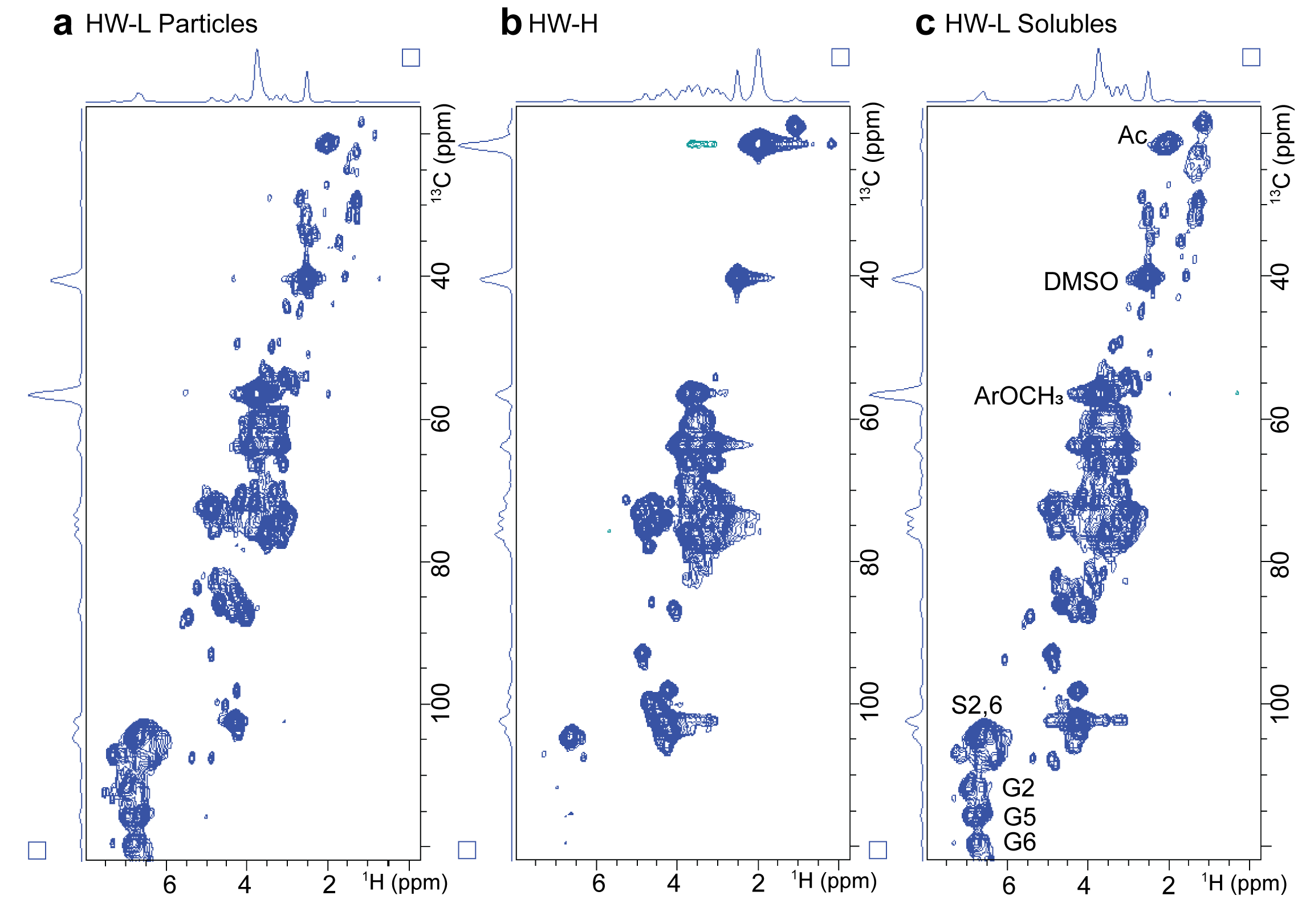
 **Figure S3**. HSQC NMR spectra of a) particle fraction of HW-L, b) hemicellulose-rich products from HW, and c) soluble fraction of HW-L.


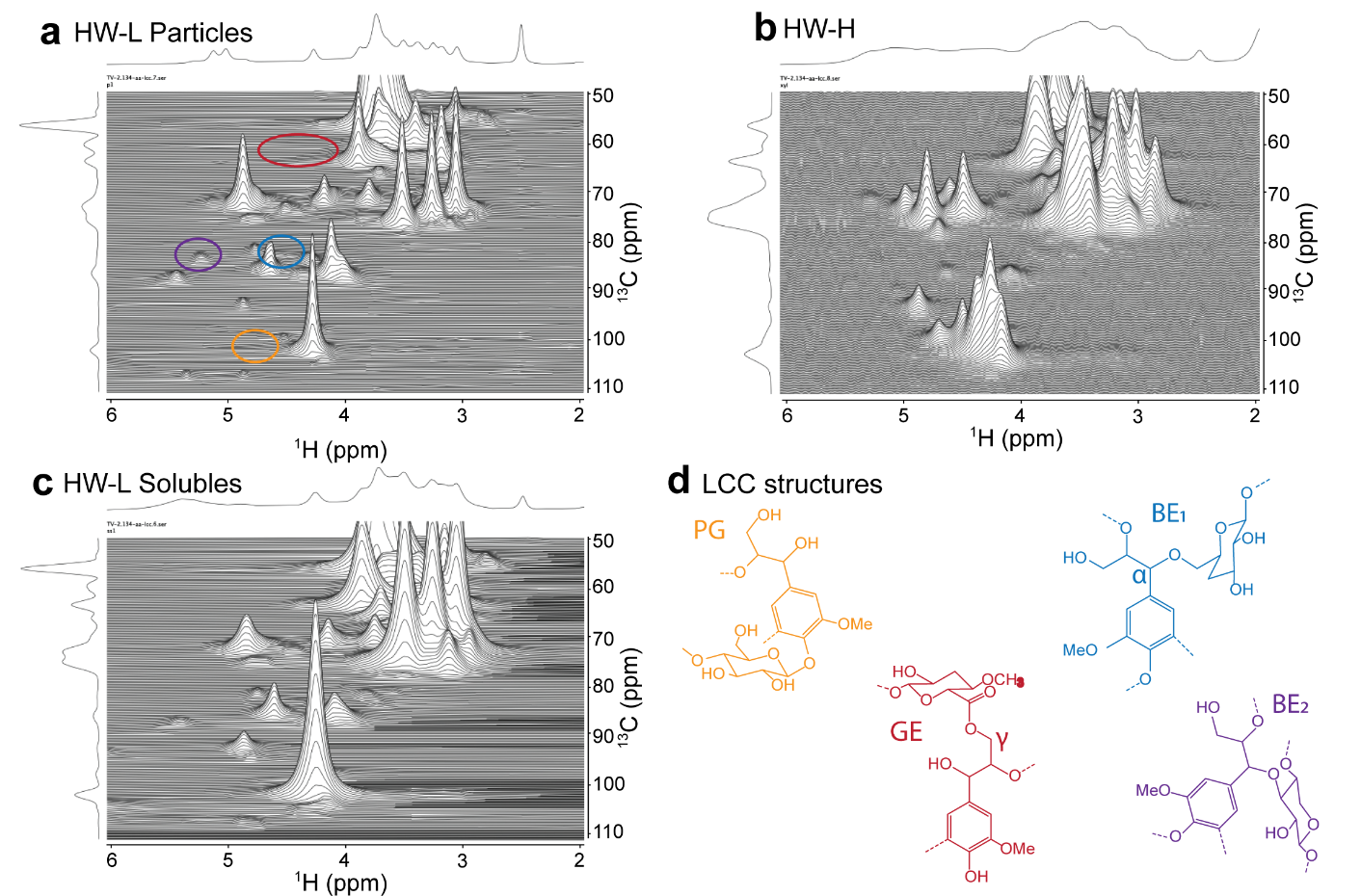
 **Figure S4.** 3D visualization of HSQC NMR spectra of **a**) particle fraction of HW-L, **b**) hemicellulose-rich products from HW, and **c**) soluble fraction of HW-L. **d**) Typical lignin-carbohydrate complex structures.


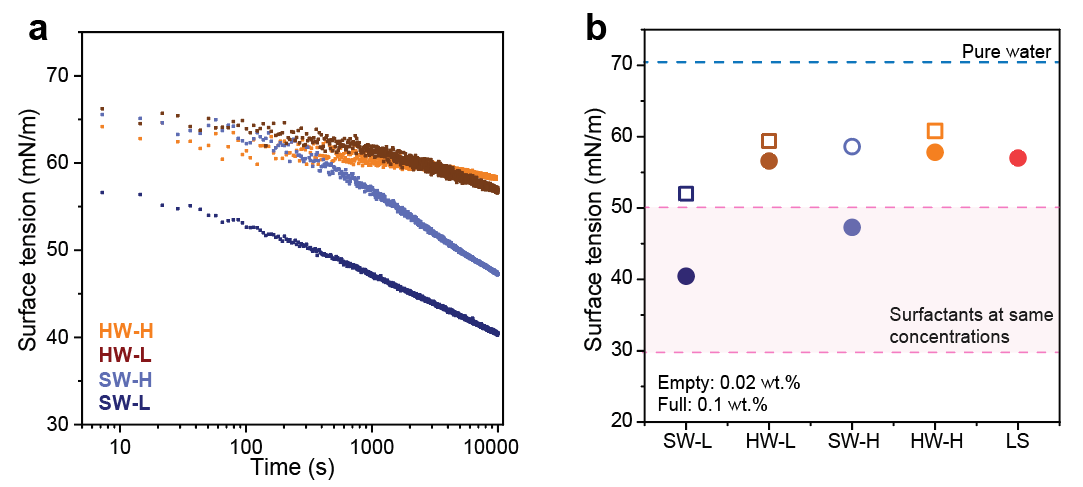


**Figure S5**. **a**) Surface tension of hemicellulose and lignin-rich fractions (from both hard and softwood) as a function of the drop surface age in seconds. **b**) Effect of the concentration of each fraction on their surface tension values, and comparison with lignosulfonate (LS) at 0.1 wt%. Note: panel **b** indicates the range values for surface tension of typical surfactants solutions used for rebounding control, e.g. sodium dodecyl sulfate (SDS), trisiloxane molecules (TSs), sodium bis(2-ethylhexyl) sulfosuccinate [Aerosol OT (AOT)]. **c**) Viscoelastic properties, acquired by dilatational rheology, of the suspensions/solutions of wood components at 0.1 wt%.

**
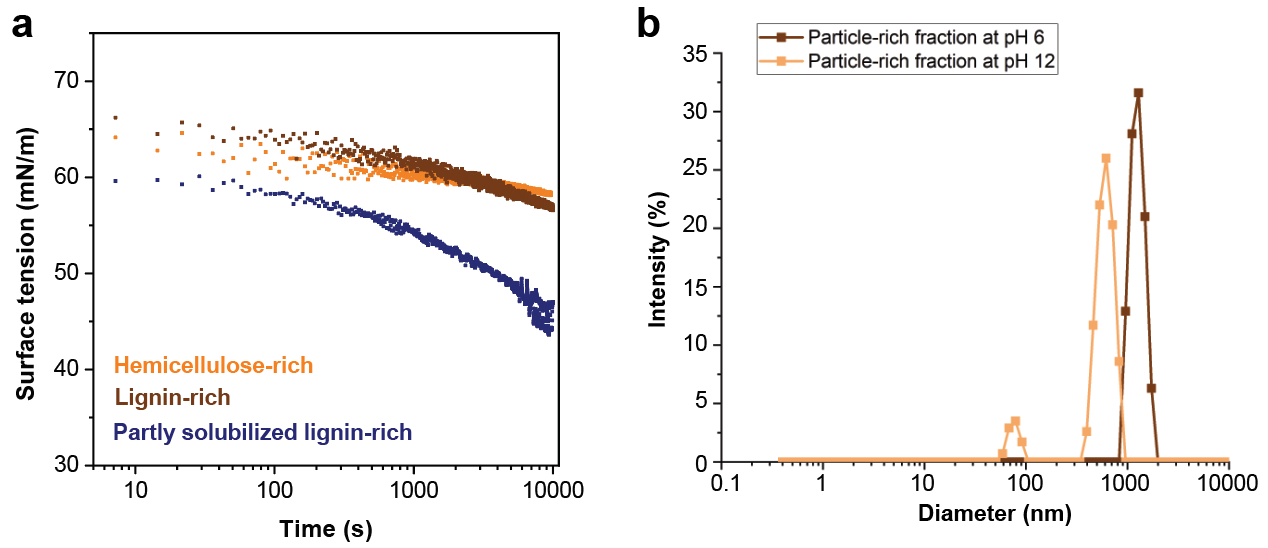
**

**Figure S6**. **a**) Dynamic surface tension profiles of HW based hemicellulose and lignin fractions as obtained, and lignin after partial solubilization. **b**) Lignin partial solubilization, at pH 12, is observed by DLS.


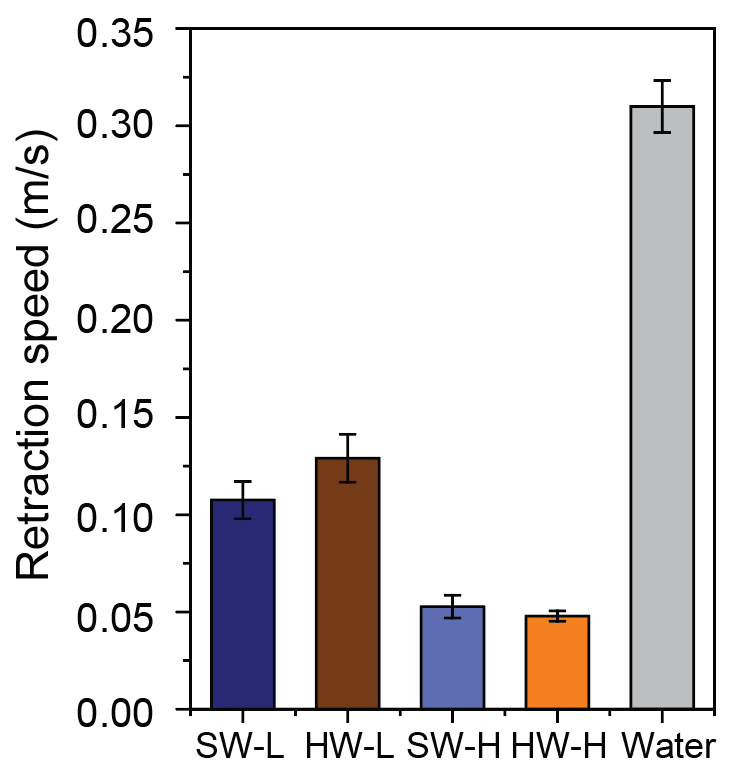


**Figure S7**. Retraction speed of droplets during the impact test on Parafilm® surface.


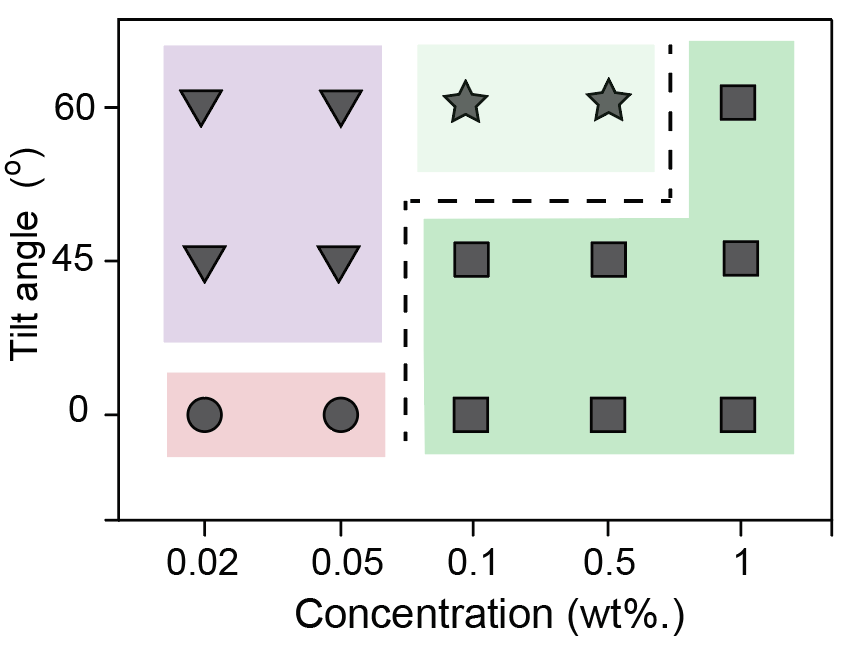


**Figure S8**. Color map of the droplet impact behavior of AOT as a function of concentration and tilting angle of the target surface. AOT: sodium bis(2-ethylhexyl) sulfosuccinate [Aerosol OT].


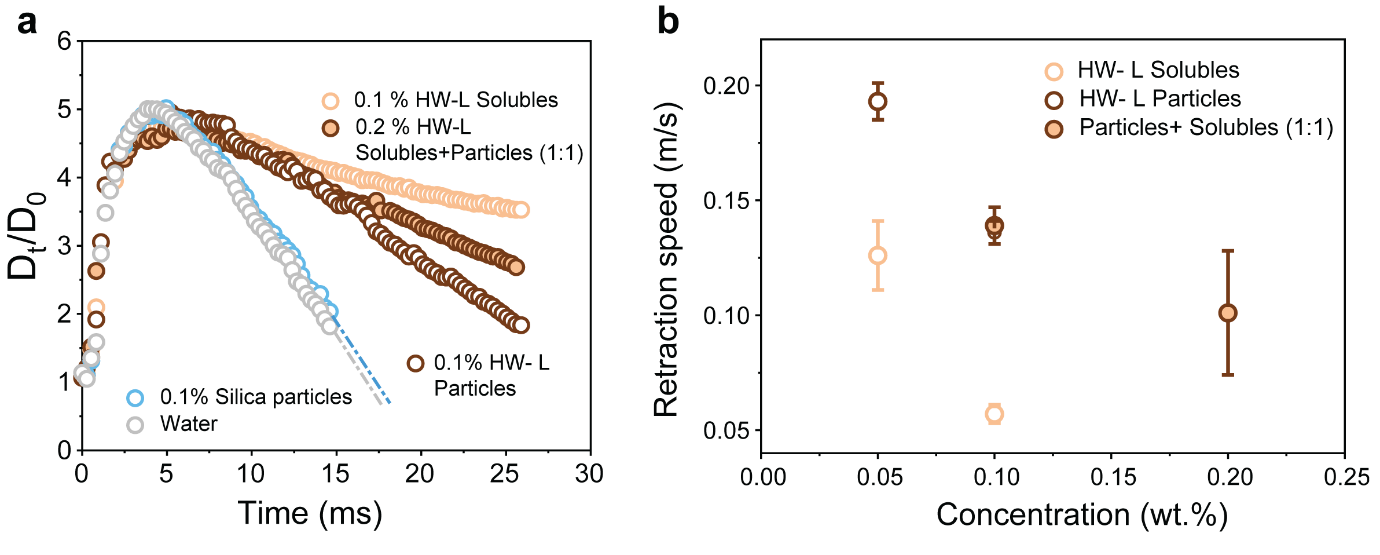


**Figure S9. a**) Dynamic droplet diameter analysis of samples during impact on Parafilm® from fractionated soluble and particle fraction from hardwood lignin-rich extracts (HW-L) at 0.1 wt.%, a mixture of these fractions at 1:1 ratio at a total 0.2 wt.%, 0.1 wt.% colloidal silica particles (~1 µm), and water as a reference. Dotted lines indicate droplet rebound. **b**) Retraction speed of fractionated soluble and particle fractions and their mixtures (1:1 ratio).


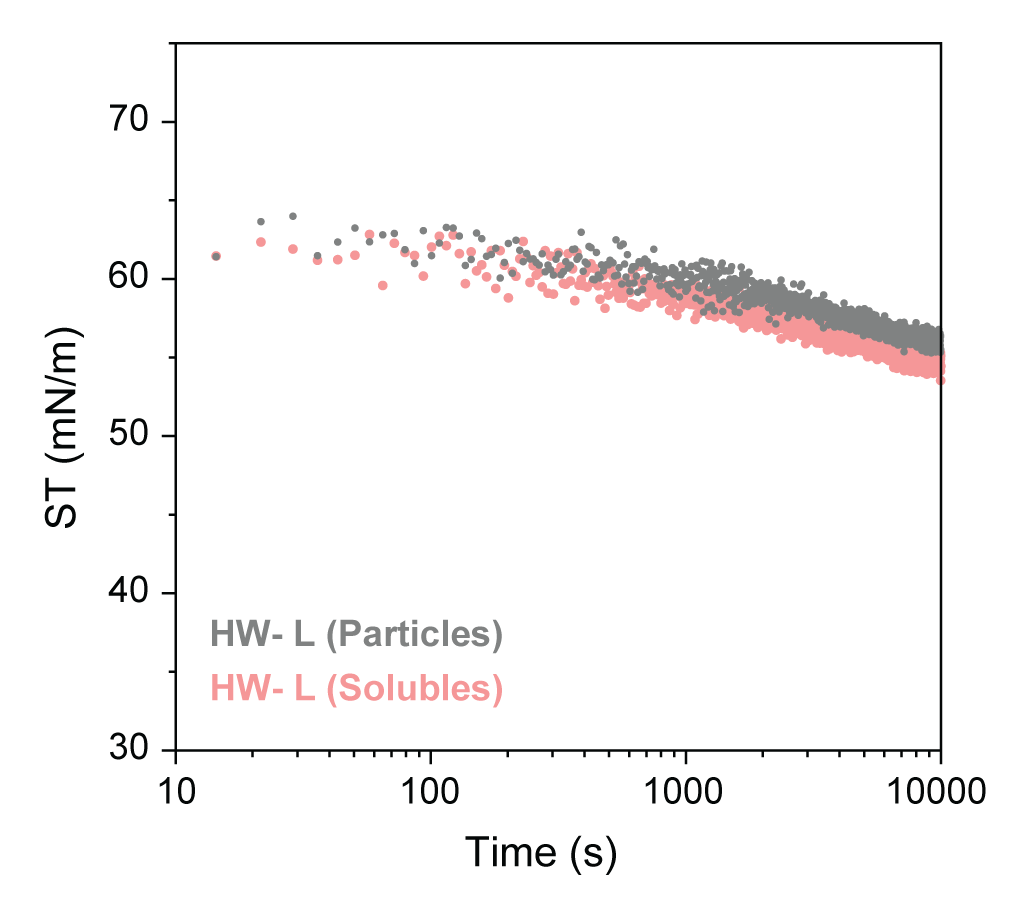


**Figure S10**. The dynamic surface tension of soluble and particle-rich fractions separated from HW-L.


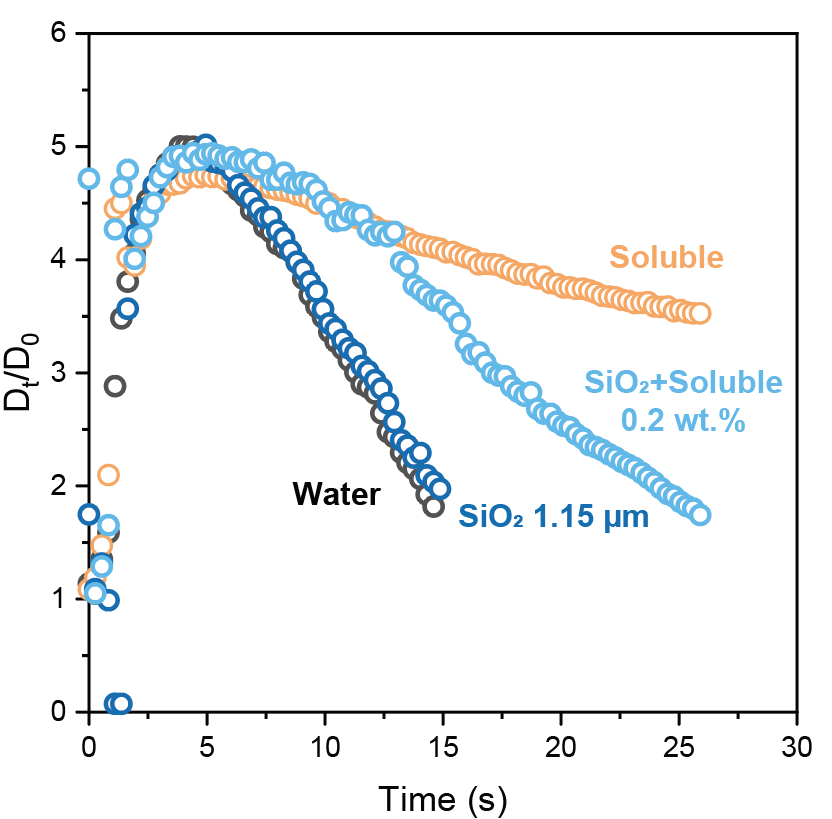


**Figure S11**. Dynamic droplet diameter analysis of samples during impact on Parafilm® from fractionated soluble fraction from hardwood lignin-rich extracts (HW-L) at 0.1 wt.%, and a mixture of these fractions at 1:1 ratio with SiO_2_ microparticles at a total 0.2 wt.%. A suspension of SiO_2_ microparticles at 0.1 wt.% and water are used as references.

**
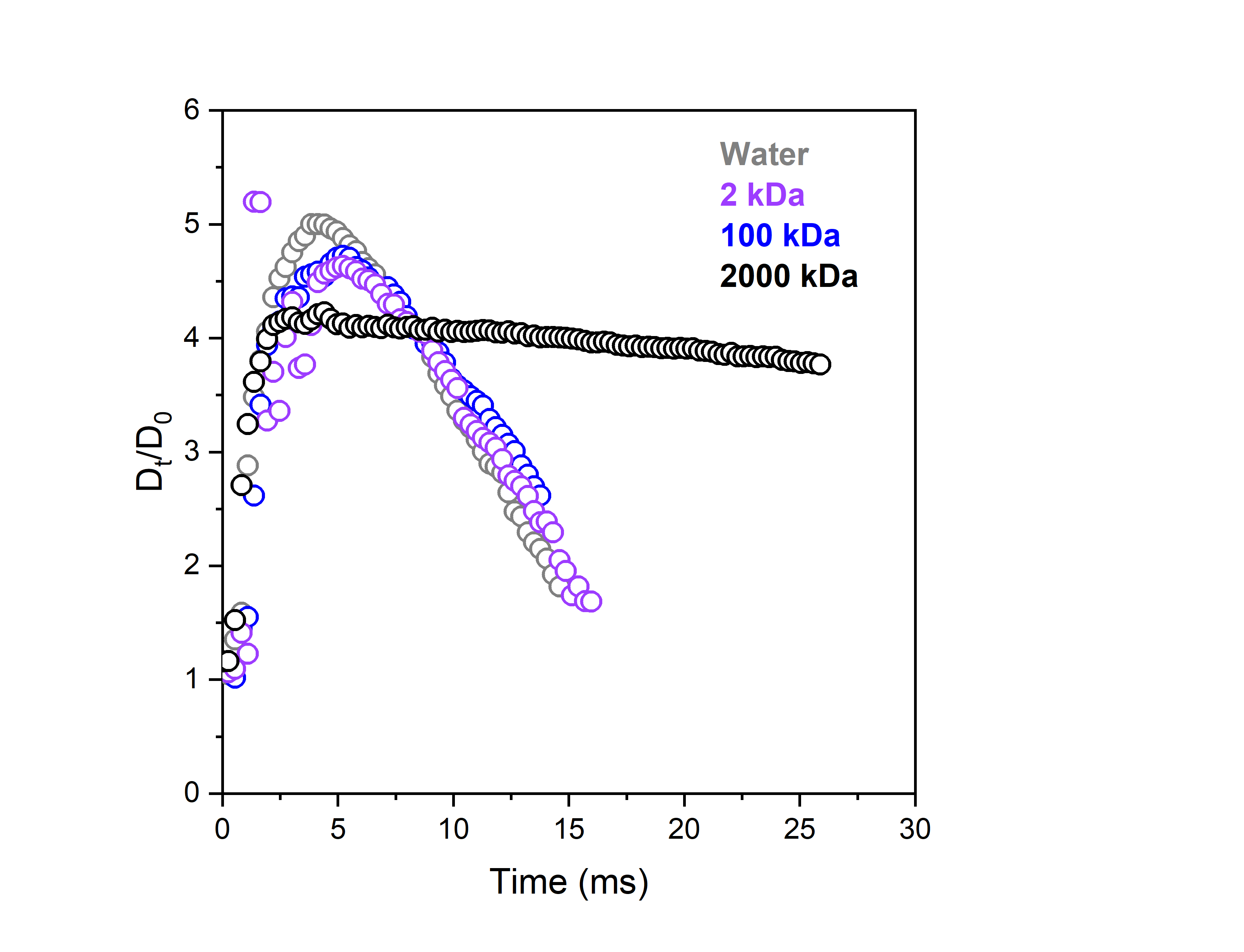
**

**Figure S12**. Dynamic droplet diameter analysis of samples during impact on Parafilm® from water and PEO solutions (at 0.1 wt.%) of varied molecular weight.


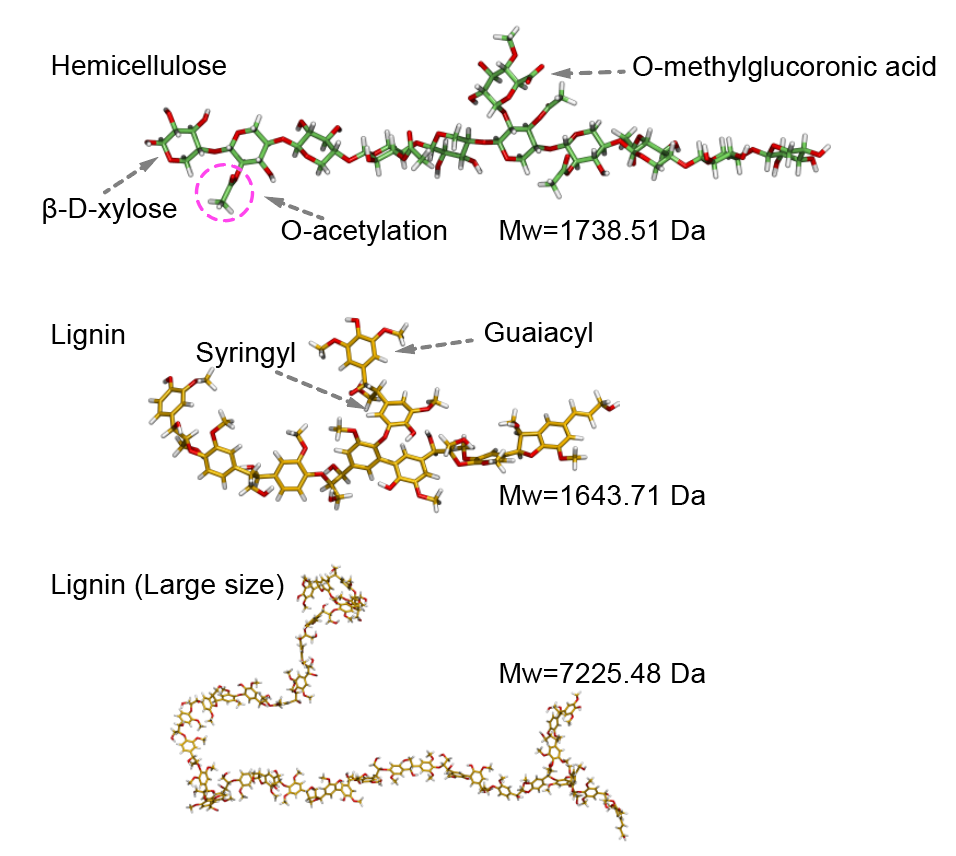


**Figure S13**. Model hemicellulose and lignin molecules used in the molecular dynamic simulations.


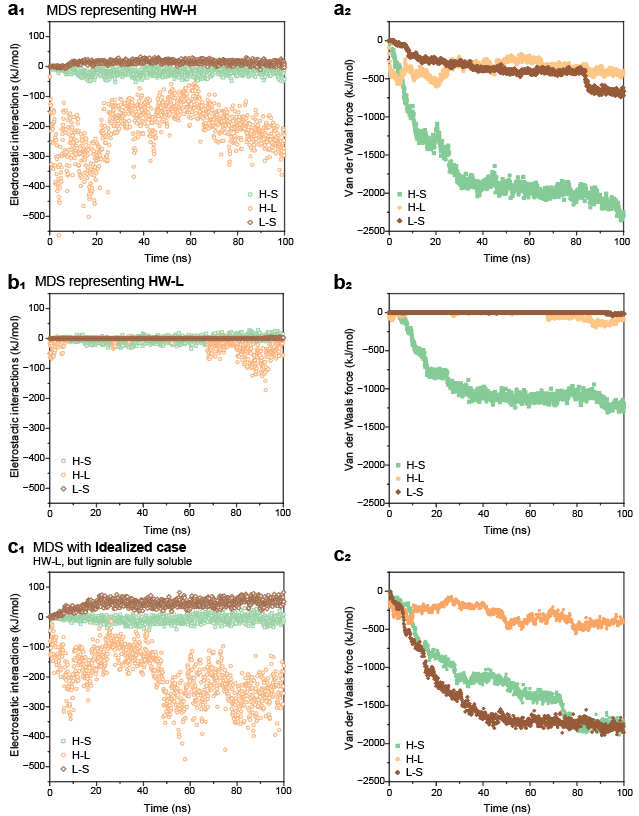
**Figure S14**. Interaction energy profiles acquired for three main interactions taking place between hemicellulose, lignin and plant surface for the a) HW-H, b) HW-H, and b) the given idealized case. Note: curves in “x1” are components from electrostatic interactions and “x2” are Van der Waals forces.


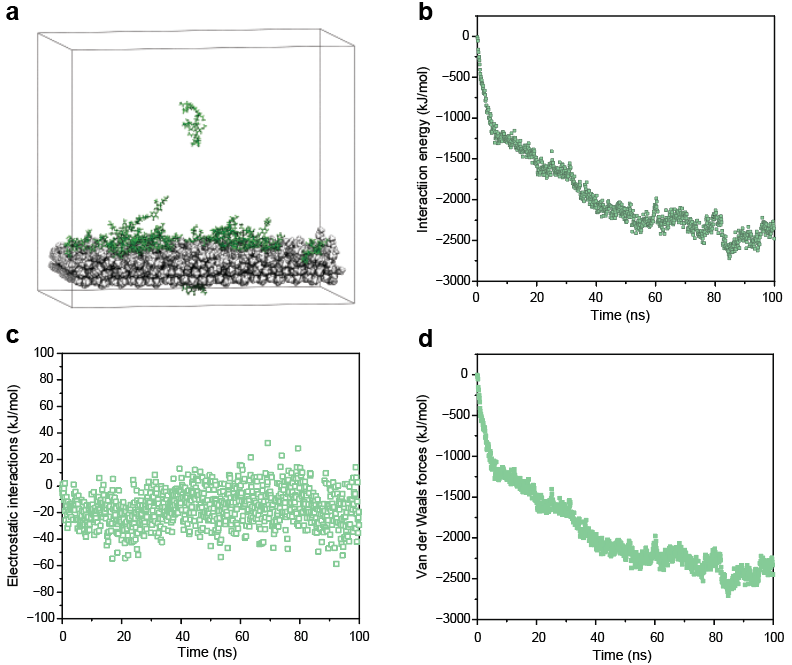
**Figure S15**. **a**) Snapshots of the molecular dynamic simulation of systems representing pure hemicellulose solutions. Interaction energy profiles acquired for three main interactions taking place between hemicellulose and plant surface, featuring **b**) total interactions energy, and **c**) electrostatic interaction and **d**) Van der Waals force components.

**
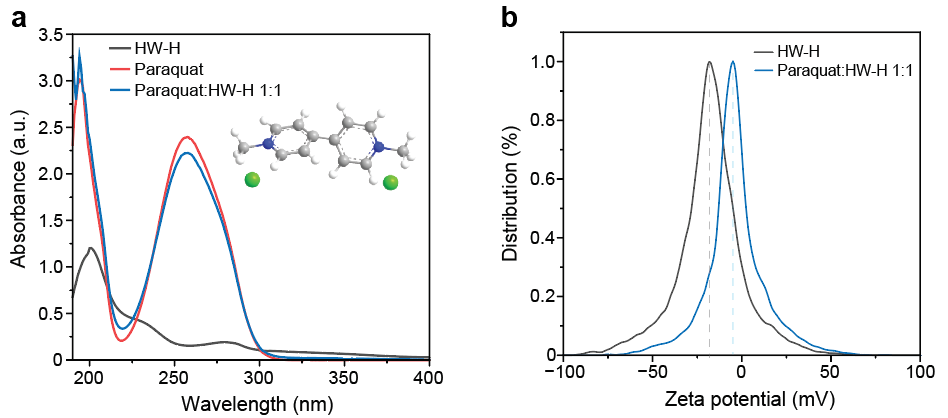
**

**Figure S16.** UV-Vis spectra (a) and zeta potential (b) of paraquat (only UV-Vis), pure HW-H and their 1:1 mixture.

**Table S1.** Chemical composition of wood fractions obtained from birch and spruce*^a^*.

| **Samples** | | **Hemicelluloses-rich extracts** | | **Lignin-rich extracts** | | **Fractionated HW-L** | |
| --- | --- | --- | --- | --- | --- | --- | --- |
|  |  | **HW-H** | **SW-H** | **HW-L** | **SW-L** | **HW-L Particle** | **HW-L**  **Soluble** |
| **Normalized chemical composition of sugars and lignins %** | GX purity *^b^* | 66.7 | 8.6 | 51.7 | 2.2 | 51.2 | 54.1 |
|  | GGM/GM *^b^* | 7.5 | 69.0 | 1.8 | 8.4 | 0.5 | 5.2 |
|  | Residual Pectins*^b^* | 7.4 | 2.0 | 2.6 | 1.6 | 1.0 | 6.2 |
|  | Insoluble lignins | 4.2 | 11.5 | 30.7 | 82.3 | 35.2 | 18.0 |
|  | Soluble lignins | 14.2 | 9.0 | 13.3 | 5.5 | 12.0 | 15.9 |
| **Normalized monosaccharide content %** | Ara | 0.9 | 0.9 | 1.3 | 8.6 | 1.0 | 1.1 |
|  | Xyl | 77.3 | 9.8 | 89.2 | 16.3 | 95.2 | 76.6 |
|  | Rha | 1.5 | 0.7 | 0.9 | 0.9 | 0.4 | 2.1 |
|  | Man | 5.4 | 61.8 | 1.9 | 45.1 | 0.6 | 4.6 |
|  | Gal | 3.9 | 9.0 | 1.3 | 10.3 | 0.2 | 3.5 |
|  | Glc | 3.8 | 16.0 | 1.3 | 13.7 | 0.4 | 3.3 |
|  | MeGlcA | 4.4 | 1.0 | 3.0 | 1.9 | 1.9 | 5.3 |
|  | GlcA | ND | ND | ND | ND | ND | 0.8 |
|  | GalA | 2.8 | 0.8 | 1.2 | 3.3 | 0.3 | 2.8 |

ND= Not detected

*^a^* In the table, HW-H and HW- L correspond to hardwood hemicelluloses-rich and ligin-rich extract, respectively, same way for softwood (SW), HW-L Particles and HW-L Solubles are fractionated parts from HW-L. Analysed monosaccharides are Arabinose (Ara), Xylose (Xyl), Rhamnose (Rha), Mannose (Man), Galactose (Gal), Glc (Glucose), 4-O-Methylglucuronic acid (MeGlcA), Glucuronic acid (GlcA) and Galacturonic acid (GalA).

*^b^* Estimated purity of glucuronoxylans (GX) calculated from content of xylose, 4-O-methylglucuronic acid. In softwoods, purity of galactoglucomannans (GGM) calculated from content of mannose, glucose, and galactose, whereas in hardwood, residual glucomannans (GM) estimated from content of mannose and glucose. Similarly, residual pectins estimated from other identified sugars: arabinose, rhamnose, galacturonic acid, and galactose (excluded in the case of softwood).

**Table S2**. HSQC NMR assignments and abbreviations.

| **Abbreviation** | **Species** |
| --- | --- |
| β–β (α) | Resinol a-CH correlation |
| β–β (β) | Resinol b-CH correlation |
| β–β (γ) | Resinol g-CH_2_ correlation |
| β–O-4 (α) | b-Aryl ether a-CH correlation |
| β–O-4 (β) | b-Aryl ether b-CH correlation |
| β–O-4 (γ) | b-Aryl ether g-CH_2_ correlation |
| β–5 (α) | Phenylcoumaran a-CH correlation |
| β–5 (β) | Phenylcoumaran b-CH correlation |
| β–5 (γ) | Phenylcoumaran g-CH_2_ correlation |
| X_1_ | Xylose CH-1 position |
| X_2_ | Non-acetylated xylose CH-2 position |
| X_3_ | Non-acetylated xylose CH-3 position |
| X_4_ | Xylose CH-4 position |
| X_5a_ | *Geminal* xylose CH_2_-5a position |
| X_5b_ | *Geminal* xylose CH_2_-5b position |
| X_1-a_(r) | Xylose reducing end a-CH-1 anomer |
| X_1-b_(r) | Xylose reducing end b-CH-1 anomer |
| X_1_(nr) | Xylose non-reducing end CH-1 position |
| X_5a_(nr) | *Geminal* xylose non-reducing end CH_2_-5a position |
| X_5b_(nr) | *Geminal* xylose non-reducing end CH_2_-5b position |
| X3_3_ | 3-acetylated xylose CH-3 position |
| X2_2_ | 2-acetylated xylose CH-2 position |
